# Evolution of Ivermectin Resistance in the Nematode Model *Caenorhabditis elegans* : Critical Influence of Population Size and Altered Emodepside Efficacy

**DOI:** 10.1111/eva.70241

**Published:** 2026-04-24

**Authors:** Jacqueline Hellinga, Barbora Trubenova, Jessica Wagner, Marc Borchert, Jürgen Krücken, Roland R. Regoes, Hinrich Schulenburg, Georg von Samson‐Himmelstjerna

**Affiliations:** ^1^ Department of Veterinary Medicine, Institute for Parasitology and Tropical Veterinary Medicine Freie Universität Berlin Berlin Germany; ^2^ Veterinary Centre for Resistance Research Freie Universität Berlin Berlin Germany; ^3^ Institute of Integrative Biology ETH Zurich Zurich Switzerland; ^4^ Zoologisches Institut Christian‐Albrechts‐Universität zu Kiel Kiel Germany; ^5^ Antibiotic Resistance Group Max‐Planck Institute for Evolutionary Biology Ploen Germany

**Keywords:** *Caenorhabditis elegans*, computational modeling, experimental evolution, ivermectin resistance, parasitic nematode

## Abstract

The emergence and spread of anthelmintic resistance represent a major challenge for treating parasitic nematodes, threatening mass‐drug administration control programs in humans and zoonotic hosts. Currently, experimental evidence to understand the influence of management (e.g., treatment dose and frequency) and parasite‐associated factors (e.g., genetic variation, population size and mutation rates) is scarce. To rectify this knowledge gap, we performed controlled evolution experiments with the model nematode *Caenorhabditis elegans* and further evaluated the evolution dynamics with a computational model. Large population size was critical for rapid ivermectin resistance evolution in vitro and in silico. Male nematode production was favored during resistance evolution, suggesting a selective advantage of sexual recombination under drug pressure in vitro. Evolution of ivermectin resistance led to reduced efficacy of the structurally related anthelmintic moxidectin, as anticipated, as well as reduced efficacy of the structurally unrelated anthelmintic emodepside, which has a distinct mode of action. In contrast, albendazole, levamisole, and monepantel efficacy were not influenced by the evolution of ivermectin resistance. We conclude that combining computational modeling with in vitro evolution experiments to test specific aspects of evolution directly represents a promising approach to guide the development of novel treatment strategies to anticipate and mitigate resistance evolution in parasitic nematodes.

## 1 Introduction

Ivermectin (IVM) belongs to the anthelmintic class of macrocyclic lactones (MLs) (Campbell 1981). It was first discovered in the late 1970s, and due to its unprecedented spectrum of efficacy and generally good safety profile, it quickly became one of the most often used pharmaceutical compounds in veterinary medicine. Subsequently, IVM was also developed for use in humans, e.g., against various filarial infections, including *Onchocerca volvulus*, the cause of river blindness (Greene et al. 1985). This use of IVM eventually led to the award of the Nobel Prize in Physiology or Medicine for its discoverers, Satoshi Omura and William C. Campbell, in 2015 (Laing et al. 2017; The Nobel Assembly at Karolinska Institutet 2015). It is still widely used against several parasitic worms in veterinary and human medicine with several pharmaceutical formulations (Crump 2017; Martin et al. 2021; Sharun et al. 2019).

In nematodes, IVM binds irreversibly to inhibitory glutamate‐gated chloride ion (GluCl) channels on membranes of (e.g., pharyngeal) muscles, motor neurons, or the reproductive tract, leading to an influx of chloride ions (Arena et al. 1995; Cully et al. 1994). This inhibition ultimately results in flaccid paralysis of the respective muscles (Crump 2017; Martin et al. 2021; Shaver et al. 2024; Wolstenholme and Rogers 2005; Yates et al. 2003). The GluCl channel consists of five membrane spanning subunits which are expressed by a species‐specific divergent number of genes. For example, in *Caenorhabditis elegans* there are six genes—i.e., *avr‐14*, *avr‐15*, *glc‐1*, *glc‐2*, *glc‐3*, and *glc‐4*. The sensitivity of the GluCl channels depends on the respective subunit composition, which has been found to differ between tissues in a given nematode species and between species (Martin et al. 2021). Dent et al. (2000) reported that mutations in two of the three GluCl genes (*glc‐1*, *avr‐14*, and *avr‐15*) in *C. elegans* led to resistance.

Since the introduction of IVM and its widespread use, resistance has been increasingly described for endo‐ and ectoparasites of veterinary importance (Bassetto et al. 2024; Cai et al. 2024; Furnival‐Adams et al. 2024; Krücken et al. 2024; Mohammedsalih et al. 2024; van Wyk and Malan 1988) and decreased IVM sensitivity has been reported in parasitic nematodes of humans such as *O. volvulus* (Doyle et al. 2017; Frempong et al. 2016; Osei‐Atweneboana et al. 2007). In parasites there are multiple pathways thought to cause IVM resistance; however, the exact mechanism of resistance is not yet known, and it may well differ between nematode species and even between different populations of the same species. Resistance to IVM was reported for *Cooperia oncophora* (*C. oncophora*) with a L256F mutation in AVR‐14 α‐type subunit of a glutamate‐gated chloride channel (Njue et al. 2004). Also, in *C. oncophora*, IVM resistance was seen when AVR‐14B was knocked out (El‐Abdellati et al. 2011). Moreover, P‐glycoproteins (Pgps) are likely to contribute to IVM resistance, as their overexpression was observed in IVM resistant *Haemonchus contortus* where the sheep were infected from a known IVM‐resistant *H. contortus* line (Mate et al. 2022). Much of the work describing the expression of parasite Pgps in IVM resistance has been done in *C. elegans* , where parasite Pgp genes are transgenically added to *C. elegans* (Janssen et al. 2015).

Genetically, IVM resistance was further linked to a locus on chromosome 5 of *H. contortus* (Doyle et al. 2019). This was subsequently confirmed by quantitative trait loci mapping of *C. elegans* lines, revealing two IVM resistance loci on chromosome 5 (Evansid et al. 2021). However, 40% of the genes in the *C. elegans* loci match the gene order of those in the *H. contortus* locus, while the rest are spread over the *H. contortus* chromosome (Evansid et al. 2021). The first locus in *C. elegans* contains one of the previously described genes, *glc‐1*, while the second locus has not been described before. Another study by Ménez et al. (2019) reported that a loss‐of‐function mutant of the nuclear hormone receptor gene *nhr‐8* in *C. elegans* resulted in sensitivity, and strains with deletions in the *nhr‐8* gene in its DNA‐binding domain also showed increased sensitivity to IVM. While many pathways are suspected to be responsible for resistance in parasites, the exact mechanism(s) and also their evolutionary emergence remain elusive. Therefore, additional research on resistance pathways and the impact of factors associated with resistance evolution, such as treatment frequency and intensity, genetic diversity, and reproductive strategies, is vital for improving our understanding of how IVM resistance evolves and, in turn, advancing sustainable parasite control methods.

The free‐living non‐parasitic nematode *C. elegans* represents a highly informative model for dissecting the molecular targets of many anthelmintics and associated resistance mechanisms. The use of *C. elegans* is appealing because it has a short development cycle of 3.5 days at 20°C, can be easily maintained on agar, has an annotated genome, and various genetic tools are available. Due to their short development cycle and the ease with which their population genetics can be manipulated, *C. elegans* has been used in evolutionary biology to answer questions about host‐pathogen interactions and the impact of outcrossing (Cutter et al. 2019; Masri et al. 2015). A study by James and Davey (2009) investigated the evolution of IVM resistance by gradually increasing IVM concentrations over multiple generations. They generated IVM‐resistant worms over 44 generations (6.9 nM IVM resistance) and 60 generations (11.4 nM IVM resistance). Their RT‐qPCR analysis revealed overexpression of Pgps and ABC transporters in the evolved resistant (6.9 and 11.4 nM IVM resistance worms, James and Davey 2009). Figueiredo et al. (2018) further applied this step‐wise resistance evolution approach to obtain a *C. elegans* strain resistant to 34.2 nM IVM. While trying to uncover the IVM‐resistance mechanism, these studies left gaps by not asking specific questions to address how individual factors (i.e., population size, genetic variation, and treatment intensity) contribute to IVM resistance.

Population biological and genetic modeling, while used extensively to understand and predict the evolution of drug resistance in viruses, bacteria, and eukaryotic microparasites (Opatowski et al. 2011), has been less commonly applied to the study of anthelmintic resistance evolution in parasitic metazoans, including nematodes. Most computational models for parasitic worms investigated the role of susceptible strain reservoirs in resistance spread (Barnes et al. 1995; Cornelius et al. 2016; Leathwick 2012; Leathwick et al. 2017; Sauermann et al. 2019). These models offer valuable insights into sustainable treatment strategies. Still, the complexities of polygenic resistance and the impact of critical factors such as ploidy, reproductive strategies, and genetic variation are less frequently addressed in existing models. However, the effect of genetic architecture, dominance, and mating preference on the rate of resistance evolution has already been emphasized (Coffeng et al. 2024; Sauermann et al. 2019; Schwab et al. 2006), highlighting the need for new, tailor‐made polygenic models of anthelmintic drug resistance evolution. A recent, computational modeling study focused on the worm's reproductive strategy explored the role of various factors, such as dominance and the mating fraction in drug resistance evolution (Trubenová et al. 2025). These computational results further suggest the importance of population size, but these findings are yet to be experimentally verified.

This study used *C. elegans* to combine experimental evolution with a proof‐of‐concept computational model to investigate mechanisms of resistance and to determine how population size as an intrinsic factor in a genetically varied worm strain undergoing sexual recombination influences the rate of IVM resistance evolution. The in vitro experimental evolution approach uses the new nematode medium named Viscous medium previously used in *C. elegans* ‐pathogen coevolution (Papkou et al. 2019, 2021) and adapted for the study of *C. elegans* resistance to anthelmintic drugs (Hellinga et al. 2024). IVM is introduced to *C. elegans* populations at increasing concentrations over time, generally following the approach by James and Davey (2009). The study reported here is the first IVM resistance evolution experiment to include biological replicates, optional male outcrossing during evolution, treatments with different population sizes, and the reporting of reduced drug efficacy to emodepside (EMO). Though the presence of males during the evolution experiment is not an independent experimental factor, the maintenance of the male proportion in the IVM‐treated lines in comparison to the control lines in the in vitro experiment is consistent with past indications that males are favored in populations in stressful environments. The results obtained by the computational model illustrating the impact of population size were supported by the in vitro experiment.

## 2 Materials and Methods

### 2.1 *Caenorhabditis elegans* Maintenance and *Escherichia coli* Preparation

**FIGURE 1:**
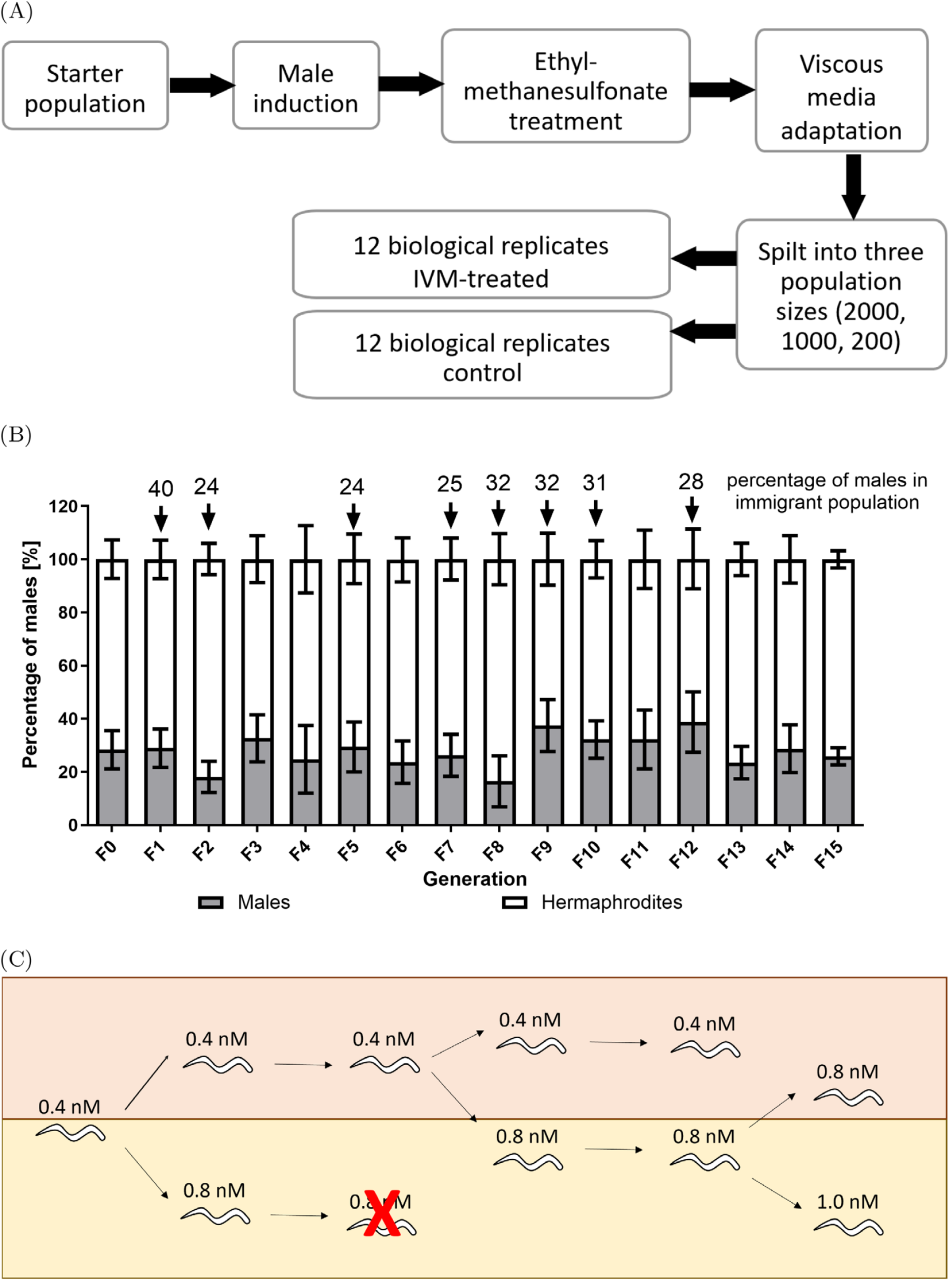
Setup of the in vitro evolution experiment with *Caenorhabditis elegans* . (A) Workflow of in vitro evolution experiment including V medium pre‐adaptation and the start of the evolution experiment. (B) Percentage of males in each generation during the V medium pre‐adaptation. The arrows indicate when immigrant males from NGM agar plates were added. The numbers above the arrows indicate the percentage of males in the immigrant population. Data show means ± standard deviation for hermaphrodite and male percentages. (C) Exposure of IVM‐treated 2000 population from generation 3–6 for the working (orange background) and challenger (yellow background) lines. Red X indicates the population did not survive the IVM concentration.

The hermaphrodite *Caenorhabditis elegans* strain WBM1133 (wbmIs63 [myo‐3p::3XFLAG::wrmScarlet::unc‐54 3′UTR *wbmIs61]) (Silva‐García et al. 2019) was obtained from the Caenorhabditis Genetics Center (CGC). The strain expresses a fluorescent red protein in its body wall muscles and was described to have a superficial wild‐type phenotype (Silva‐García et al. 2019). The worm strain was used as it provides a useful platform for future investigation of resistance transfer during mating between resistant and sensitive lines, and males were more frequently observed to be maintained in this strain than in N2. Worms were maintained on nematode growth medium (NGM) agar supplemented with *E* *scherichia coli* OP50 as a food source at 16°C using standard manipulation methods (Hope 1999). An overview of the workflow for the evolution experiment is provided in Figure 1A. Males were introduced into the population by seeding an NGM plate with 10 fourth stage larvae (L4) that were heat shocked at 35°C for 2 h. The male population was maintained by picking at a propagation ratio of 3:1 (male: hermaphrodite) at 20°C. *Escherichia coli* OP50 was prepared as described in Hellinga et al. (2024).

### 2.2 Ethyl Methanesulfonate Treatment

Synchronized male containing WBM1133 worms were resuspended in M9 buffer (42.5 mM Na_2_HPO_4_, 22.0 mM KH_2_PO_4_, 85.5 mM NaCl, 1 mM Mg_2_SO_4_). Worms were incubated in 50 mM of EMS dissolved in M9 under constant shaking at 20°C for 4 h. Worms were collected by centrifugation and washed five times with 15 mL of M9. As previously described in Brenner (1974). An F2 screen was set up to assess the reproductive health of the EMS‐treated worms. Ten gravid hermaphrodite worms from EMS‐treated and non‐treated populations were placed on NGM agar with one worm per plate. The gravid worms were removed after 24 h, and the resulting offspring were counted (Figure S1). These counts were plotted in GraphPad Prism (v. 10.5.0) showing the mean and standard deviation and a paired‐*t*‐test was performed.

### 2.3 Pre‐Adaptation to Viscous Medium

The experimental evolution was performed in a Viscous medium (V medium) a liquid medium with increased viscosity. V medium was chosen to ensure even drug exposure during treatment with IVM; standard nematode growth medium (NGM) agar can result in heterogenous drug distribution, and axenic liquid media have been reported to lead to delayed and distorted development compared with growth on agar. Further details on the properties of V medium can be found in Hellinga et al. (2024) and Papkou et al. (2019). Pre‐adaptation of *C. elegans* to V medium was done to ensure the worm populations were adapted to the main conditions of the experimental approach, and the V medium did not act as an additional selective factor during the main experiment (Papkou et al. 2019). In the pre‐adaptation process 3.2 mL of Viscous medium (S basal (10 mM potassium phosphate buffer pH 6.0, 100 mM NaCl), 0.8% hydroxypropyl methylcellulose, 10 mM potassium citrate pH 6.0, 3 mM Mg_2_SO_4_, 3 mM CaCl_2_, 5 μg/mL cholesterol) was placed into each well of a 6‐well culture plate (Sarstedt #83.3920, Sarstedt AG and Co. KG, Nümbrecht, Germany) supplemented with *E. coli* OP50 (final optical density, OD_600_ = 2.0). Worms were incubated at 20°C with constant shaking at 150 rpm for 3–4 days. The worms were pooled at each transfer and washed with 200× volume of S basal. The pooling method used at each transfer was done as previously described in Hellinga et al. (2024). At each transfer the size of the worm population was counted in 15 10 μL drops and set to 3000 worms per well. In total, 15 transfers were done; at transfers 1, 5, 7, 8, 9, 10, and 12, a male‐rich immigrant population that had undergone EMS treatment was added as 10% of the total population to maintain the percentage of males during the adaptation at 30% (Figure 1B). These male‐rich immigrant populations were maintained on NGM with a male: hermaphrodite ratio of 4:1. To add further genetic variation, at transfer 2, male induced WBM1133 worms that didn't undergo EMS treatment from the Schulenburg Lab, Kiel University, Germany were added to the population corresponding to 16% of the total worm population. For each transfer, 15 drops (10 μL each) were mixed with 1 μL 5% Lugol's iodine to count the number of males in the population (Figure 1B). Only L4 and older worms were counted, and the worm was determined to be a male if it had either the characteristic male tail or lacked 2 gonad arms or eggs. After the 15th transfer, worms were pooled and divided into 8 batches containing 22% males. These populations were synchronized by bleaching, and the corresponding 8 batches of L1 worms were counted and divided into replicates and allowed to evolve independently in the evolution experiment as described below.

### 2.4 The Evolution Experiment

Synchronized L1 worms from the V medium adaptation were divided into 72 biological replicates for the evolution experiment. Half of the populations (*n* = 36) were used for selection with IVM (IVM‐treated lines), and the other half was used as a control (control lines) for genetic drift in the absence of IVM. For each of these two treated lines, 12 replicates were used for each population size. The population sizes were set to 200, 1000, and 2000 worms. The control populations were exposed to 0.1% dimethyl sulphoxide (DMSO) for the evolution experiment. The IVM‐treated populations were exposed to IVM in a step‐wise manner. The IVM concentrations started with 0.1 nM and were doubled to 1.0 nM; all following concentration steps were 25%–33% higher than the last (Figure S2). All populations were grown in 12‐well culture plates (Sarstedt #83.3920, Sarstedt AG and Co. KG, Nümbrecht, Germany); each well contained 1.6 mL V medium supplemented with *E. coli* OP50 (final optical density OD_600_ 1.3 for 2000 worms, 1.0 for 1000 worms, and 0.5 for 200 worms). Worms were incubated at 20°C with constant shaking at 150 rpm for 4 days. On the fourth day, the worm lines were washed with S basal, and 10 drops (5 μL each) were counted to determine the worm concentration by the calculation of worm/L (Figure S3). All lines were reset to the original population size and again incubated at 20°C. The percentage of males was determined for each population replicate as described above. To ensure that the IVM‐treated line was not lost during the evolution experiment it was divided into two groups and grown in parallel, with one group of the IVM‐treated worms exposed to the introduced concentration of IVM (working group) and the other exposed to a greater concentration of IVM (challenge group) (Figure 1C). All data and analyses presented in this study were derived exclusively from the working group. Once all working groups acquired resistance to a specific concentration of IVM, they were kept at that concentration for one more generation so each biological replicate could be expanded to freeze multiple vials for later analysis. The challenge group then became the working group, and a new challenge group at a higher IVM concentration was established (Figure 1C). If resistance was not acquired for the challenge group, then the challenge group was discarded, and propagation continued from the working group. In the absence of resistance, populations exposed to IVM are expected to show progressive loss of motility and declining population size over time. A population was only stated to have acquired resistance to the respective IVM concentration once all 12 biological replicates produced a population size 10 times higher than the starting population, and the worms were seen to move freely (Figure S3), a threshold established during initial assay setup. Finally, the 1000 worm populations were grown for 38 generations, and the 2000 and 200 worm populations were grown for 39 generations.

### 2.5 In Silico Evolution Experiment

Our proof‐of‐concept computational model used to simulate the evolution experiment was based on the population genetic, compartmental model proposed in (Trubenová et al. 2025) with a few modifications, as explained in the Supporting Information. The model considered IVM resistance a polygenic trait, with six independent diploid loci (genes) of various effects on the level of resistance. Note that these six loci do not correlate directly with any region in the *C. elegans* genome. This generated $3^{6}=729$ possible genotypes, providing substantial complexity to reflect the diverse resistant strains observed in experimental settings while remaining computationally tractable (Figure S4). Resistance mutations were assumed to be loss of function mutations and thus were recessive, meaning both copies of a gene had to be mutated for resistance to increase (see Trubenová et al. 2025 for the effect of dominance). In addition, a trade‐off was incorporated: mutations increased IVM resistance (benefit) but lowered fitness in the worms in a drug‐free environment (cost). The benefits were based on experimental measurements of drug resistance of various strains obtained from the literature (see Table S2), but the costs were determined arbitrary due to lack of available information (see Table S3), assuming correlation between the cost and benefit, with more beneficial mutations being more costly. Both benefits and costs of multiple homozygous mutations combined additively. We used empirically supported pharmacodynamic relationships between the worm fitness and the IVM concentration to determine the fitness of various genotypes under increasing IVM concentrations (see Figure S4).

The simulations mirrored the steps of the in vitro experiment (explained above), starting with susceptible *C. elegans* populations of different sizes (200, 1000, 2000) containing 20% males. The simulations of the evolutionary process were then run for a set number of cycles, corresponding to generations. In each generation, hermaphrodites mated with a probability of 10% or self‐fertilized, generating new genotypic combinations assuming no linkage among the loci. Mutations occurred at a small rate (0.0001 mutations per genome), generating new variants.

If the population grew above a given threshold (30‐fold increase), it was considered adapted, and the IVM concentration was increased (Table S3), mimicking the experimental protocol. At the end of each cycle, populations were diluted to the original size. Simulations were stochastic and repeated 100 times for each population size over 40 generations following the process outlined earlier. In addition, the same evolutionary scenario was modeled for a longer time of 80 generations, with stochastic simulations repeated 50 times for each population size. See Supporting Information for a detailed description of the simulation process and simulated scenarios (Figures S5–S14, Tables S1, S2, and S4). All simulations were run in Python. The simulation code, as well as code for the analysis is available at Github (https://github.com/Trubenova/EvolutionOfIvermectinResistance).

### 2.6 Larval Development Assays

Frozen ancestor and endpoint (generations 38/39) evolved worms were thawed and regenerated for two generations without drug pressure in V medium, as described above for adaptation to V medium and in Hellinga et al. (2024). Adults from the F2 generation were synchronized by bleaching. The L1 worms were counted in 5 5 μL drops, and approximately 100 worms were added in 24 μL to every well of a 48‐well microtiter plate already containing 200 μL V medium, 25 μL *E. coli* OP50 (OD_600_ = 2), and 1 μL anthelmintic. Albendazole (ABZ), EMO, IVM, monepantel (MON), and moxidectin (MOX) were dissolved in 100% DMSO, while levamisole (LEV) was dissolved in S basal. IVM was added in a serial dilution to the final concentration range of 0–8 nM IVM with a 1.4‐fold dilution across 15 concentrations for the control and ancestor populations and 0–110 nM IVM with a 1.4‐fold dilution across 15 concentrations for the resistant populations. For MOX, a 1.5‐fold dilution was done across 15 concentrations with the final concentration range of 0–6 nM MOX for the control and ancestor populations and 0–75 nM MOX for the resistant populations. EMO had a 1.4‐fold dilution across 15 concentrations with a final concentration range of 0–100 nM EMO for the control and ancestor populations and 0–600 nM for the resistant populations. The drugs ABZ had a final concentration range of 0–100 μM ABZ across 15 concentrations with a 1.5‐fold dilution, MON had a final range of 0–100 nM MON across 15 concentrations with a 1.4‐fold dilution, and LEV had a final range of 0–250 μM LEV across 15 concentrations with a 1.6‐fold dilution. Worms were incubated at 20°C with constant shaking at 150 rpm. After a 48–52 h incubation, the development was stopped by adding 5% Lugol's iodine. Developed worms were either L4 or adult stage (males and hermaphrodites), while non‐developed worms were L1, L2, and L3. For each resistant/control population, a biological replicate of the assay was performed once with anthelmintic drug concentrations in triplicate. The ancestor population was used as a control in all experiments. The mean EC_50_ values were determined by fitting a variable slope log ([IVM]) vs. response (percentage developed worms) four‐parameters logit curve using GraphPad Prism 10.5.0.

### 2.7 Statistical Analyses

#### 2.7.1 Larval Development Assay

The mean EC_50_ values with 95% confidence intervals (Cis) were determined by fitting a variable slope log ([IVM]) vs. response (percentage developed worms) four‐parameter logit curve using GraphPad Prism 10.5.0. Significant differences between the mean EC_50_ values were determined by the extra sum of squares F‐test within GraphPad Prism. *p* < 0.05 were considered to be statistically significant. For development analysis, significant differences in development between the ancestor and the endpoint evolved lines were calculated using a non‐parametric Kruskal–Wallis test followed by a Dunn's post hoc test comparing the evolved lines to the ancestor.

#### 2.7.2 In Vitro Evolution Experiment

ANOVA was used to identify significant differences in the percentage of males during the in vitro evolution experiment, followed by a Tukey's Multiple Comparison post hoc test in GraphPad Prism.

#### 2.7.3 Genetic Mutation Rate

Genome resequencing samples of the ancestor and final evolved populations were prepared by thawing and growing the worms in V medium as described above for larval development assays. The synchronized worms were grown in population sizes of 3000 in 6‐well culture plate (Sarstedt AG and Co. KG, Nümbrecht, Germany) supplemented with *E. coli* OP50 (OD_600_ = 1.0) without exposure to IVM at 20°C. After 48 h the worms were washed and stored at −80°C for DNA isolation. DNA isolation was done with the Macherey–Nagel NucleoSpin Tissue kit (MACHEREY‐NAGEL GmbH and Co. KG., Düren, Germany). Library preparation and genome resequencing were performed by BioMarker Technologies (Münster, Germany). A processed variant call format (VCF) file from the genome resequencing was used in this analysis. To estimate de novo mutation rates, we processed VCF data using R (v4.4.2) and the vcfR (v 1.15.0), dplyr (v 1.1.4), and tidyr (v. 1.3.1) packages. The VCF file was imported using read.vcfR(), and genotype data were extracted into a matrix format with extract.gt(). Chromosome and position information were appended to the genotype data, which was then reshaped to long format to associate each sample with its corresponding genotype. Samples were annotated by a predefined experimental group. To focus on derived variation, the missing data and homozygous reference genotypes (i.e., “0/0”, “./.”, and “.”) were filtered out. Ancestral mutations were provided either from the VCF file and were removed from the evolved sample data using an anti‐join operation to isolate de novo variants. Mutation counts were then aggregated per experimental group and used to calculate mutation rates, assuming a genome size of 100 Mb and 39 generations of evolution. Final results were saved as a CSV file.

## 3 Results

### 3.1 Population Size Increases the Rate of IVM Resistance Evolution During the In Vitro Evolution Experiment

**FIGURE 2:**
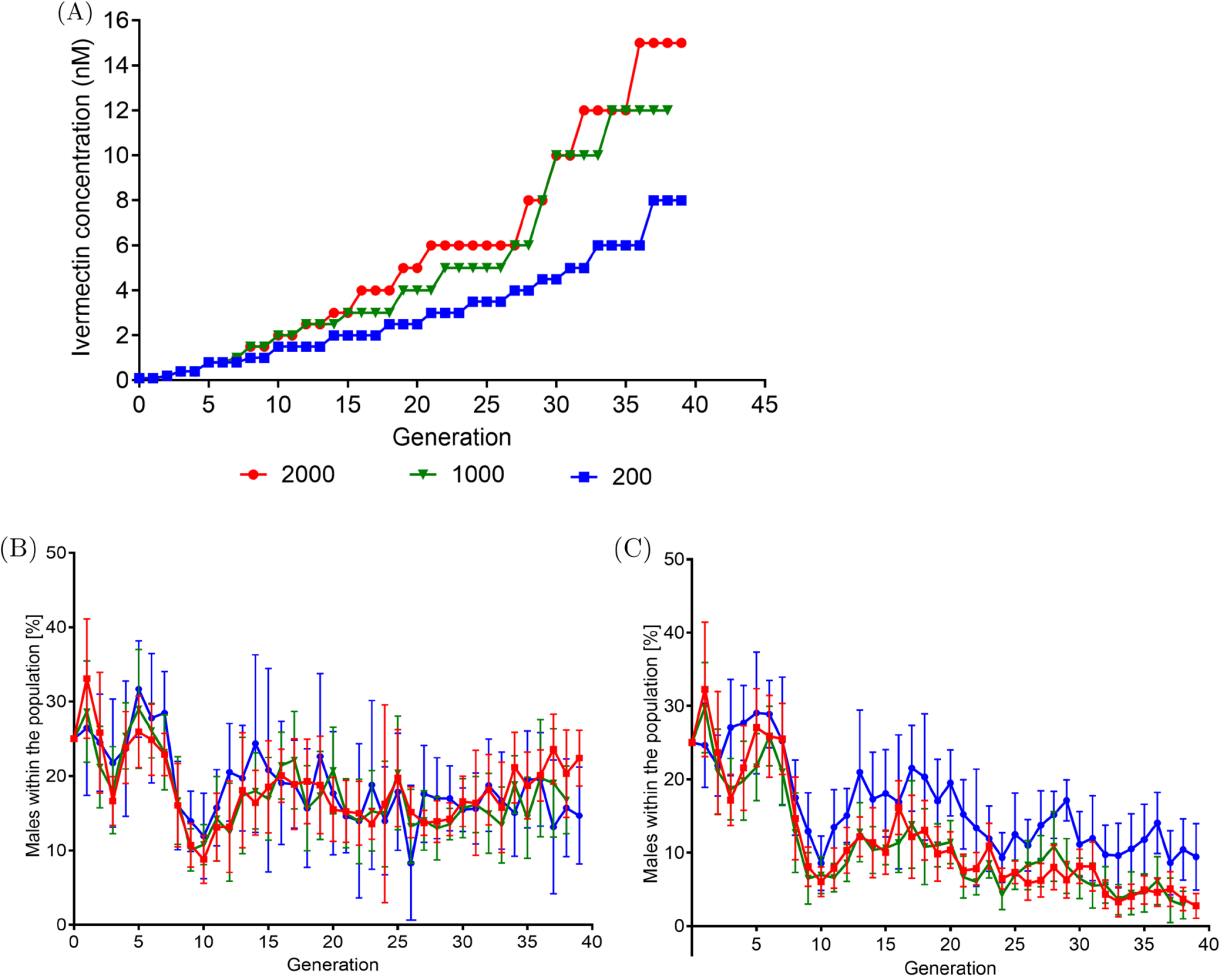
Outcome of the in vitro evolution experiment. Population sizes are indicated by red squares (2000), green triangles (1000) and blue circles (200). (A) Concentration of IVM tolerated by *Caenorhabditis elegans* populations during the in vitro evolution experiment. (B, C) Percentage of males found in IVM‐treated lines (B) and control lines (C). Graphs show the mean (solid lines) and the standard deviation (error bars) of the 12 biological replicates. Adult stage/L4 stage worms were counted in 10 10 μL drops. Worms were paralyzed with Lugol's reagent so that anatomical structures could be observed.

For the in vitro evolution experiment, synchronized worms adapted to V medium were divided into three population sizes: 200, 1000, and 2000 worms, with 12 biological replicates for both control and IVM‐treated lines. Worms were exposed to increasing IVM concentrations (Figure S2). When introduced to 0.8 nM IVM, the 200‐worm populations took longer to evolve resistance than the 1000 and 2000 populations. Throughout the experiment, the IVM‐treated line took either two to three generations to adapt to a given IVM concentration and were not considered to be adapted until the population size was 10 times higher than the starting population size. However, at one point during the evolution experiment, each IVM‐treated line required more than three generations to adapt to an IVM concentration. This was observed for the 2000 IVM‐treated line when it was introduced to 6.0 nM IVM, the 1000 IVM‐treated line at 5.0 nM IVM, and the 200 IVM‐treated line at 2.0 nM IVM. Lastly, smaller IVM increments were used for the 200 IVM‐treated line to prevent extinction. The experiment was initially planned for 40 generations but was concluded early when the 1000 IVM‐treated line adapted to 12 nM IVM by generation 38, and the 2000 and 200 IVM‐treated lines adapted to 15 nM and 8 nM IVM, respectively, by generation 39 (Figure 2A). The results demonstrated that population size influenced the rate of IVM resistance evolution.

### 3.2 IVM Selection Led to a Significantly Higher Frequency of Males in All Population Sizes

Initially, males were introduced to the *C. elegans* population to ensure a possibility for outcrossing as a basis for maintaining genetic variation and to minimize the number of deleterious mutations (Chasnov and Chow 2002) within the biological replicates for each population size. Males were not an independent factor during the evolution experiment as they were initially present at a similar proportion in all evolving lines. In the IVM‐treated lines, males were easily maintained throughout the evolution experiment. In the first 10 generations, the percentage of males was similar in both control and IVM‐treated lines (Figure 2B,C). The similarity may be due to the fact that the concentration of IVM to which the worms were exposed was below the threshold known to induce stress based on previous phenotypic assays (Dent et al. 2000; Hellinga et al. 2024). At generation 11, the percentage of males in the IVM‐treated lines remained stable at approximately 20%, while the percentage in the control lines slightly increased but overall decreased over time (Figure 2B,C).

Interestingly, the percentage of males in the final generation of the IVM‐treated lines decreased with smaller population sizes: the 2000 IVM‐treated line had the highest percentage of males at 22.4%, the 1000 IVM‐treated line had 16.8%, and the 200 IVM‐treated line had the lowest at 14.7% (Figure 2D). The differences in the percentage of males between IVM‐treated and control populations were statistically significant (*p* < 0.001, One‐way ANOVA, Tukey's multiple comparison test) (Table 1). The higher percentage of males in the 200 control line compared to the 1000 and 2000 control lines could be an artifact due to fewer worms in the L4/adult stage being counted.

**TABLE 1:** Percentage of males observed in the endpoint generation of the in vitro evolution experiment. The *p* values were determined by one‐way ANOVA and Tukey's multiple comparison post‐test comparing the percentage of males between the resistant and control populations.

Line	2000R	2000C	1000R	1000C	200R	200C
Percentage	22.4 ± 3.7	2.7 ± 1.7	16.8 ± 4.5	2.8 ± 1.8	14.7 ± 6.5	9.4 ± 4.5
*p*	< 0.0001		< 0.0001		< 0.0001	

### 3.3 Computational Modeling Shows the Impact of Population Size on Both Evolutionary Rate and Trajectory

We developed a computational model to investigate if the experimental observations are consistent qualitatively and quantitatively with the population biology and genetic characteristics of the system, such as population sizes, the mutation rate of *C. elegans* , and its pharmacodynamics. In a nutshell, the model describes the reproduction and evolution of the worms in the experiment, assuming six diploid resistance loci (see Materials and Methods). The computational model used in this work was based on a framework previously developed by Trubenová et al. (2025) to understand the role of the various mating systems of *C. elegans* as well as of other factors, including population size, in resistance evolution to anthelmintics.

**FIGURE 3:**
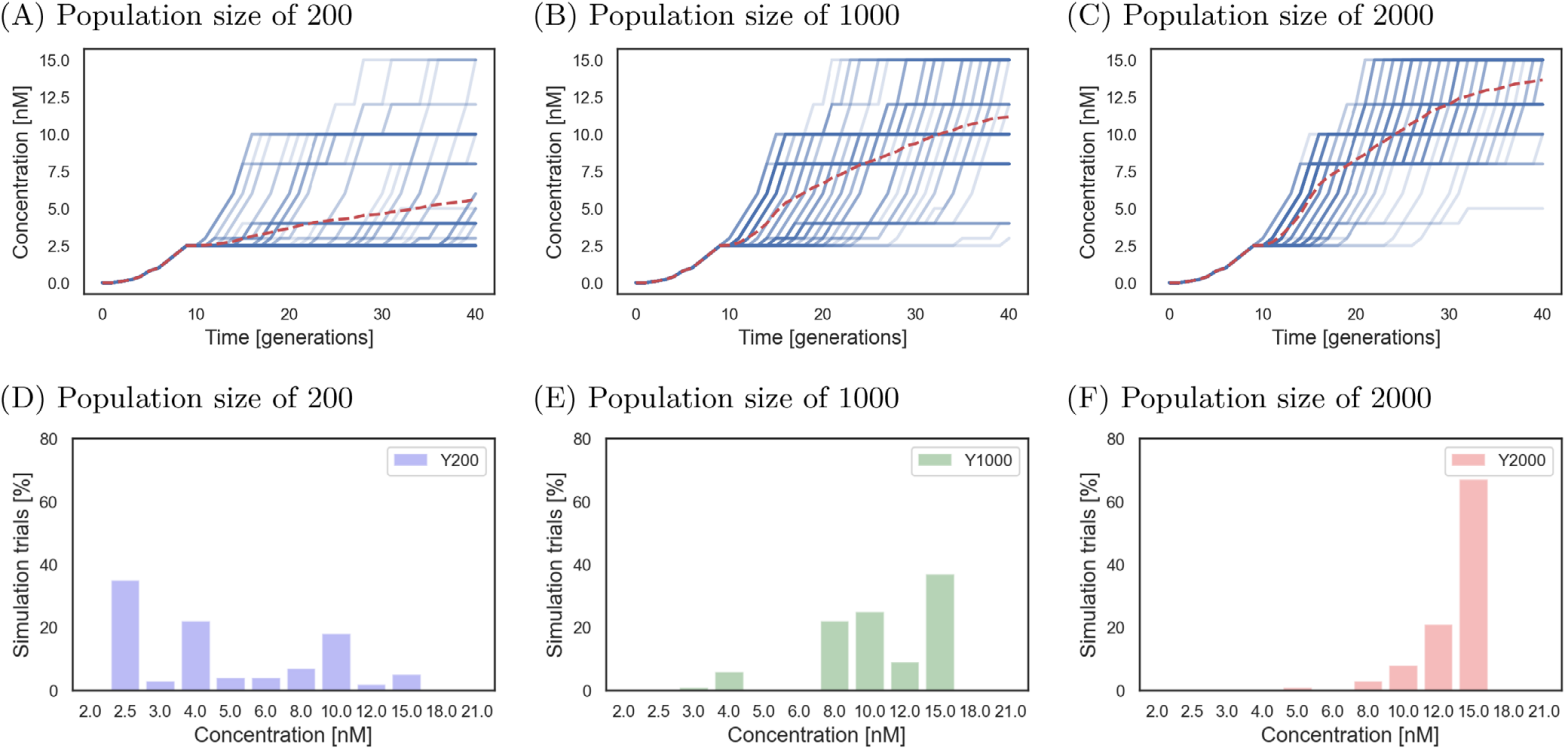
Computational model of three *Caenorhabditis elegans* population sizes evolving under IVM over 40 generations. The concentration at which the worm populations grew as a function of time in the simulated evolution experiments (A–C). Blue solid lines correspond to individual simulations (*n* = 100); the red dashed lines represent the means. Distribution of the final IVM concentrations of the *C. elegans* populations adapted after 40 generations (D–F). (A, D) population size 200, (B, E) population size 1000, (C, F) population size 2000.

**FIGURE 4:**
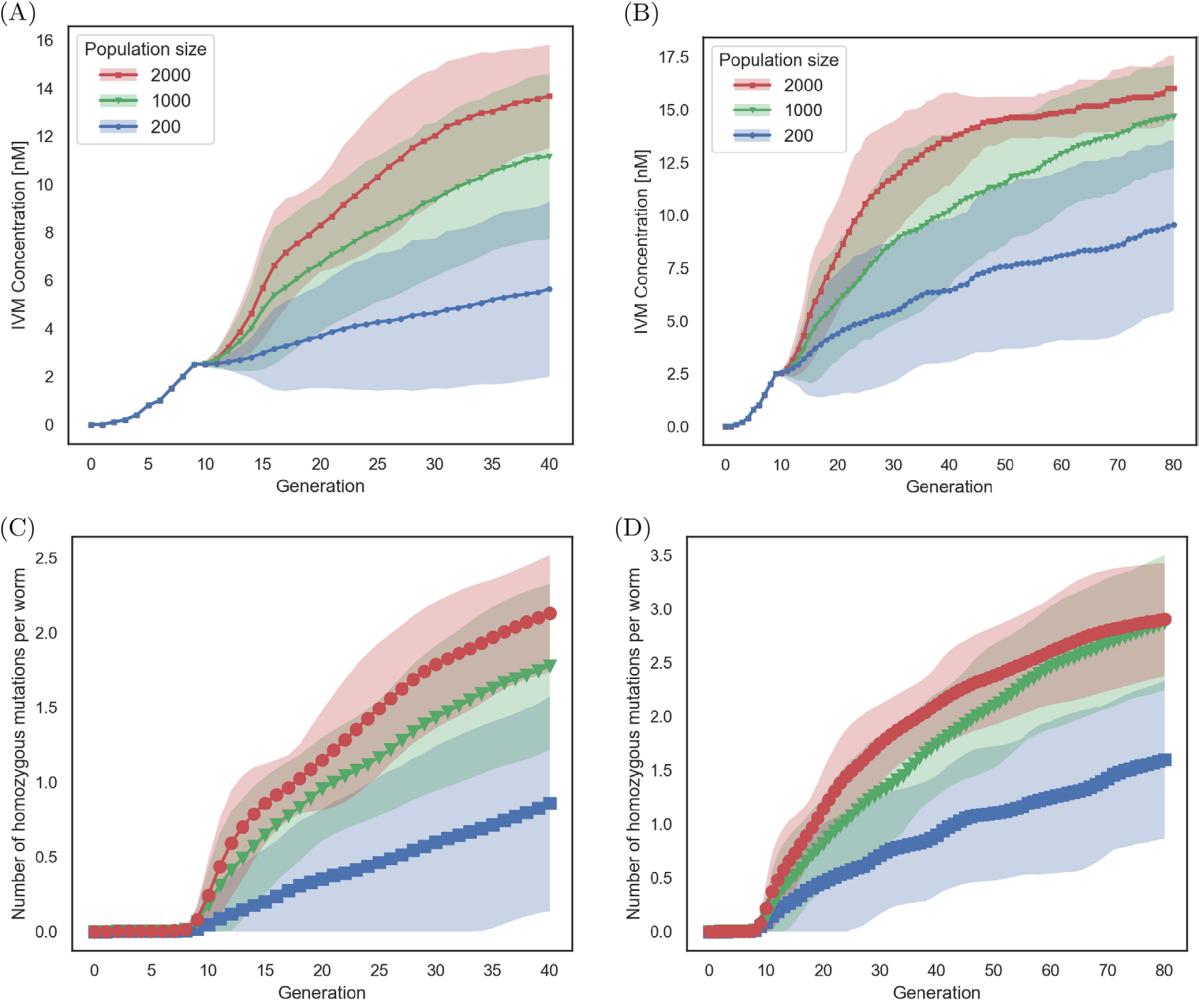
The IVM concentration range the *Caenorhabditis elegans* populations adapted to over time and the number of homozygous mutations predicted to occur while adapting. The 200 population are shown in blue, 1000 in green, and 2000 in red. (A, B) Modeling results for concentrations at which the worm populations are cultured as a function of time. (A) 40 generations and (B) 80 generations. The line represents the mean, and the standard deviations are the shaded areas. (C, D) The average number of homozygous mutations per worm present within the population at a certain time point: (C) 40 generations and (D) 80 generations, mean (lines) and standard deviation (shaded areas).

As in the in vitro evolution experiment, our model shows a clear advantage for larger populations in the rate of resistance evolution. This reflects the experimental finding that the 2000 population size outpaced the 1000 and 200 population sizes in adapting to increasing IVM concentrations. In addition, the simulations revealed several important insights into the evolution of IVM resistance:

Firstly, we observed that all populations adapted to low IVM concentrations (up to 2.0 nM) within the first nine generations. The EC_50_ of the computational model for the initial population was set to 1 nM IVM to reflect literature values for wild‐type *C. elegans* (Table S2). Therefore, even the wild‐type worms can reproduce sufficiently at these concentrations, with the average egg count above 30, a set threshold needed to progress to higher concentrations (Figure 3A). However, individual simulations showed varying outcomes at higher concentrations. While some populations failed to adapt beyond 2.0 nM IVM, others, even those with small population sizes, adapted to 15 nM IVM within 40 generations. This highlights the impact of random mutations, which become especially significant in smaller populations. Larger populations showed more consistent adaptation trajectories, as they had a higher supply of beneficial mutations (Figure 3B,C). In agreement with experimental observations, the mean (Figure 4A) and distribution (Figure 3D,E) of the final IVM concentrations reached different levels between population sizes. Lastly, the model agreed with the mutation rate calculated from the final evolved lines which showed that the largest IVM‐treated population size had the highest mutation rate (Table S1).

In the extended simulations of 80 generations, smaller populations (200 and 1000) adapted to higher IVM concentrations (Figure 4B). Interestingly, while the largest population (2000) also reached, on average, resistance to a higher concentration, its median adaptation (15 nM IVM) remained similar to the smaller populations (15 nM IVM for population of 1000 worms, 10 nM IVM for populations of 200 worms, Figure S11). This suggests that while initial adaptation is more likely in larger populations due to increased mutation supply, the difference between 1000 and 2000 individuals may not be large enough to impact long‐term adaptation rates significantly. While the average number of homozygous mutations increased over time in all population sizes (Figure 4C,D), as stated above, none of the populations reached a state in which all loci would be mutated and homozygous. This is because at the reached concentration of 15 nM IVM, it is the triple mutant (with three loci of large effect mutated) that is the fittest.

**FIGURE 5:**
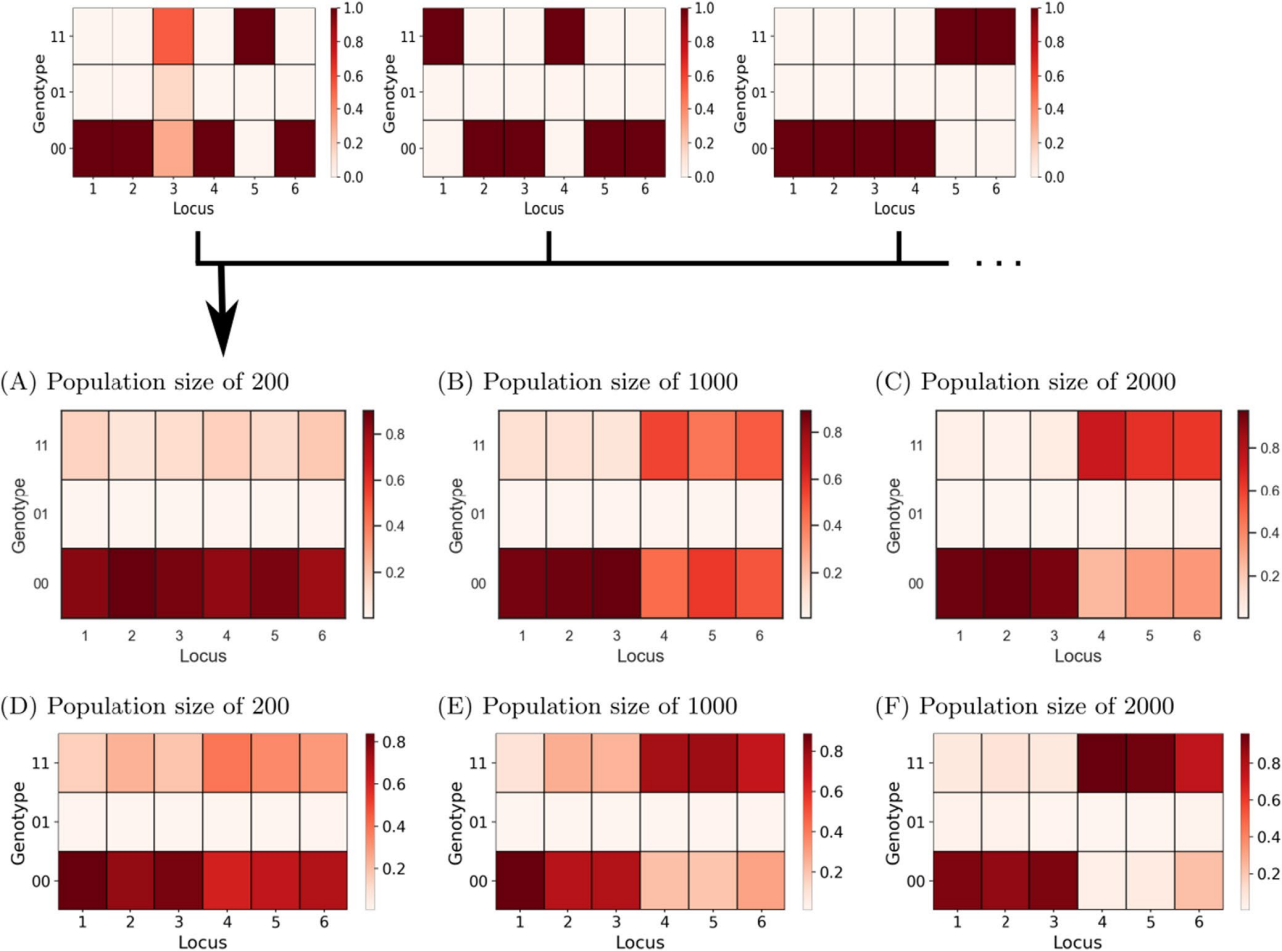
Genetic composition of the population at the end of the in silico evolution experiment, mean of 100 individuals simulations. Different loci are shown on the *X*‐axis, and the possible genotypes for each locus are shown on the *Y*‐axis. The rectangle's color corresponds to the fraction of individuals with a particular genotype at that locus. Values in each column add to 1. (A–C) Genetic composition of the final populations reached after 40 generations of evolution. (D–F) Genetic composition of the final populations reached after 80 generations of evolution. (A, D) 200 populations, (B, E) 1000 populations, (C, F) 2000 populations.

To deepen our insight into the outcome of the evolutionary process, we investigated in silico the genetic composition of the final populations after 40 generations of evolution. Our simulations revealed substantial variation in the genetic makeup of resistant populations (Figures S7–S9). Population size had an impact on which resistance loci were fixed in the population (Figure 5). In small populations (200 individuals), any resistance mutation had a similar chance of becoming fixed, likely due to the limited mutation supply (Figure 5A). However, in larger populations (1000 and 2000), mutations with larger effects were more likely to dominate (Figure 5B,C). This suggests that clonal interference (competition between strains with different beneficial mutations), rather than mutational supply, plays a role in these larger populations. Although the 6 loci generated in the mathematical model do not correspond to a specific region in the genome of *C. elegans* , observations of the mutation rate for the evolved worm lines by the mathematical model were still confirmed by genomic resequencing of our evolved and ancestor populations. In the larger 2000 and 1000 IVM‐treated lines, we saw a higher frequency of mutations in comparison to their corresponding control lines (Table S1). Remarkably, the 200 IVM‐treated line does not have a higher frequency of mutation than its control line, and the frequency was also lower than for both the 2000 and 1000 IVM‐treated lines.

In populations that evolved over 80 generations, a clear preference for mutations with larger effects emerged over time, even in the smallest populations (Figure 5D–F, Figure S14). Additionally, in the largest population, simulations revealed selection against mutations with the highest cost (locus 6), favoring those with equal benefits but lower costs (loci 4 and 5) (Figure 5F). Combining this insight with the previous observations, we hypothesize that while the mutations of three large‐effect loci were sufficient to allow populations to adapt to 15 nM IVM, the beneficial effects of the remaining three loci were too weak to allow further adaptation. In addition, fitness costs accumulated, preventing further adaptation.

### 3.4 Emergence of Reduced Drug Efficacy of the In Vitro IVM‐Adapted Populations to the Macrocyclic Lactones IVM and Moxidectin

The final generation of each biological replicate from all IVM‐treated and control lines was characterized phenotypically by larval development assays to assess the level of evolved IVM resistance across the evolution experiment. Ancestor populations were also tested, i.e., the 15th generation of the pre‐adaptation process to the V medium. All 12 biological replicates from IVM‐treated and control lines were tested for each population size.

**FIGURE 6:**
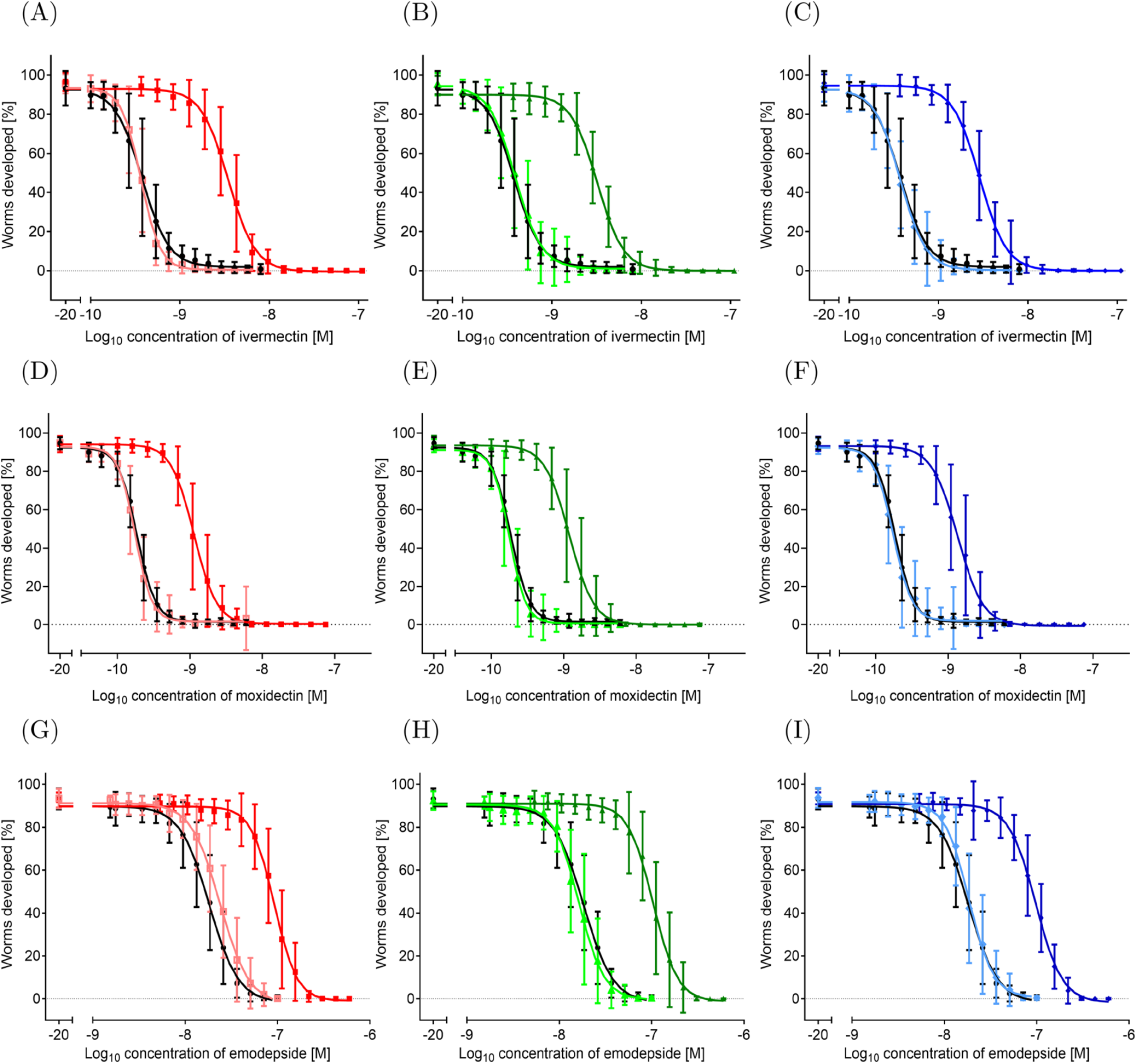
Concentration response curves profiling the responses of the ancestor and the endpoint of the IVM‐treated and control lines in an in vitro larval development assay. Ancestor is black square, the 2000 IVM‐treated line (2000R) are shown as red squares, the 2000 control line (2000C) as salmon squares, the 1000 IVM‐treated line (1000R) as dark green triangles, the 1000 control line (1000C) as light green triangles, the 200 IVM‐treated line as dark blue diamonds, and the 200 control line as light blue diamonds. (A–C) IVM, (D–F) MOX, (G–I) EMO. Plotted are the mean (solid lines) and standard deviation (error bars) of the 12 population replicates for each worm line. One biological and three technical replicates were done for each population replicate.

The 2000 IVM‐treated line had a mean EC_50_ value of 3.48 nM IVM, which is 9.40 times higher than the ancestor's EC_50_ value of 0.37 nM IVM (Table 2; Figure 6A). The 1000 IVM‐treated line had a mean EC_50_ value of 3.21 nM IVM, 8.67 times higher than the ancestor (Table 2, Figure 6B). The 200 IVM‐treated line had a mean EC_50_ value of 2.88 nM IVM, 7.78 times higher than the ancestor (Table 2, Figure 6C). Thus, all IVM‐treated lines were significantly more resistant to IVM than the ancestor (*p* < 0.0001). The fold change of resistance compared to the ancestor decreased as population size decreased. The control lines of the evolution experiment were also assessed for their resistance level to IVM. The 2000, 1000, and 200 control lines had mean EC_50_ values of 0.36 nM IVM, 0.39 nM IVM, and 0.37 nM IVM, respectively (Table 2). These mean EC_50_ values were not statistically different from the ancestor's EC_50_ values (*p* values: 0.3337 for 2000, 0.336 for 1000, and 0.860 for 200). This suggests that the conditions of the evolution experiment did not favor resistance evolution and that resistance was derived from the step‐wise introduction of IVM (Figure 6A–C). All population sizes for each IVM‐treated and control line had similar IVM EC_50_ values, indicating no outlier biological replicate.

**TABLE 2:** Mean EC_50_ values of the IVM resistant lines to MLs, IVM, and MOX as determined by larval development assays.

Anthelmintic	Population	EC_50_ (nM)	95% CI	*R* ^2^	Fold change^a^	*p* ^b^
IVM	Ancestor	0.37	0.35–0.39	0.910	1.00	
	2000R	3.48	3.34–3.63	0.938	9.40	< 0.0001
	2000C	0.36	0.35–0.37	0.943	0.97	0.337
	1000R	3.21	3.10–3.32	0.953	8.67	< 0.0001
	1000C	0.39	0.37–0.41	0.893	1.05	0.336
	200R	2.88	2.78–2.99	0.953	7.78	< 0.0001
	200C	0.37	0.35–0.39	0.917	1.00	0.860
MOX	Ancestor	0.19	0.18–0.19	0.963	1.00	
	2000R	1.23	1.17–1.28	0.930	6.47	< 0.0001
	2000C	0.17	0.17–0.18	0.928	1.00	0.010
	1000R	1.25	1.18–1.32	0.905	6.57	< 0.0001
	1000C	0.18	0.17–0.19	0.910	0.94	0.127
	200R	1.37	1.30–1.45	0.923	7.21	< 0.0001
	200C	0.18	0.17–0.19	0.901	0.94	0.060

^a^ Calculated as EC_50_ resistant or control populations/EC_50_ ancestor.

^b^ Sum of squares *F* test comparing resistant or control populations to the ancestor.

The ancestor and the endpoint of the IVM‐treated and control lines were also tested against MOX, another ML, to assess drug efficacy. The ancestor population had an EC_50_ value of 0.19 nM for MOX (Table 2). The 2000 IVM‐treated line had a mean EC_50_ value of 1.23 nM MOX, 6.47 times higher than the ancestor (Table 2, Figure 6D). The 1000 IVM‐treated line had a mean EC_50_ value of 1.25 nM MOX, which was 6.57 times higher than the ancestor (Figure 6E). Further, the 200 IVM‐treated line was the most resistant to MOX with a mean EC_50_ value of 1.37 nM MOX, which was 7.21 times higher than that of the ancestor (Figure 6F). All IVM‐treated lines were significantly more resistant to MOX than the ancestor (*p* < 0.0001 for all population sizes). The fold change in MOX resistance relative to the ancestor varied across IVM population‐size treatments, with the 200 IVM‐treated line showing the highest fold change and the 2000 IVM‐treated line showing the lowest. The control lines had mean EC_50_ values of 0.18 nM MOX for the 1000 and 200 lines (Table 2). The 2000 control line (EC_50_ of 0.17 nM MOX) was slightly more sensitive to MOX than the ancestor (*p* = 0.010), while the other control lines were not significantly different. Finally, all population replicates for the control and IVM‐treated lines had similar MOX EC_50_ values (Table 2), confirming no outlier biological replicate.

### 3.5 Emergence of Reduced Drug Efficacy of the In Vitro IVM‐Adapted Populations to Emodepside and Sensitivity to Other Anthelmintic Classes

The evolved populations were further tested against EMO, a cyclooctadepsipeptide. The ancestor had an EC_50_ value of 18.27 nM EMO (Table 3). The 2000 IVM‐treated line had a mean EC_50_ value of 91.91 nM EMO, 5.03 times higher than the ancestor (Table 3). The 1000 IVM‐treated line had a mean EC_50_ value of 103.4 nM EMO, 5.65 times higher than the ancestor (Table 3). Lastly, the 200 IVM‐treated line had a mean EC_50_ value of 97.49 nM EMO, 5.33 times higher than the ancestor (Table 3). Therefore, all the IVM‐treated lines, which also evolved resistance to MOX, showed comparable resistance to EMO (Figure 6G–I). This reduced drug efficacy to EMO was statistically significant (*p* < 0.0001, sum of squares *F* test). Egg‐to‐adult development assays in *C. elegans* have shown that EMO delays development at low‐nanomolar concentrations (Bull et al. 2007). By contrast, the fold change in EMO EC_50_ relative to the ancestor did not vary across IVM population‐size treatments. For the 2000 and 1000 control lines, slightly but significantly higher EC_50_ values (2000: *p* < 0.0001, 1000: *p* = 0.0006) were observed compared to the ancestor, while no significant difference was detected for the 200 control line (Table 3). No outlier replicate was observed as all population replicates for each IVM‐treated and control line had similar EC_50_ values (Table 3). Therefore, all the IVM‐treated lines also evolved resistance to MOX and had reduced drug efficacy to the unrelated anthelmintic EMO.

**TABLE 3:** Mean EC_50_ values of evolved IVM resistant lines to the cyclooctadepsipeptide EMO and benzimidazole ABZ as determined by larval development assays.

Anthelmintic	Population	EC_50_ (nM)	95% CI	*R* ^2^	Fold change^a^	*p* ^b^
EMO	Ancestor	18.27	17.50–19.07	0.927	1.00	
	2000R	91.91	88.71–95.22	0.932	5.03	< 0.0001
	2000C	23.95	22.93–25.01	0.925	1.31	< 0.0001
	1000R	103.4	99.04–107.9	0.912	5.65	< 0.0001
	1000C	16.38	15.68–17.11	0.912	0.89	0.0006
	200R	97.49	94.29–100.8	0.950	5.33	< 0.0001
	200C	18.68	17.95–19.44	0.922	1.02	0.4573
ABZ	Ancestor	2950	2790–3120	0.934	1.00	
	2000R	3190	3030–3350	0.936	1.08	0.04
	2000C	3410	3020–3850	0.836	1.15	0.02
	1000R	3300	3160–3450	0.952	1.11	0.002
	1000C	2730	2520–2950	0.895	0.92	0.10
	200R	2590	2480–2700	0.951	0.87	0.0002
	200C	2540	2400–2690	0.930	0.86	0.0002

^a^ Calculated as EC_50_ resistant or control populations/EC_50_ ancestor.

^b^ Sum of squares *F* test comparing resistant or control populations to the ancestor.

**FIGURE 7:**
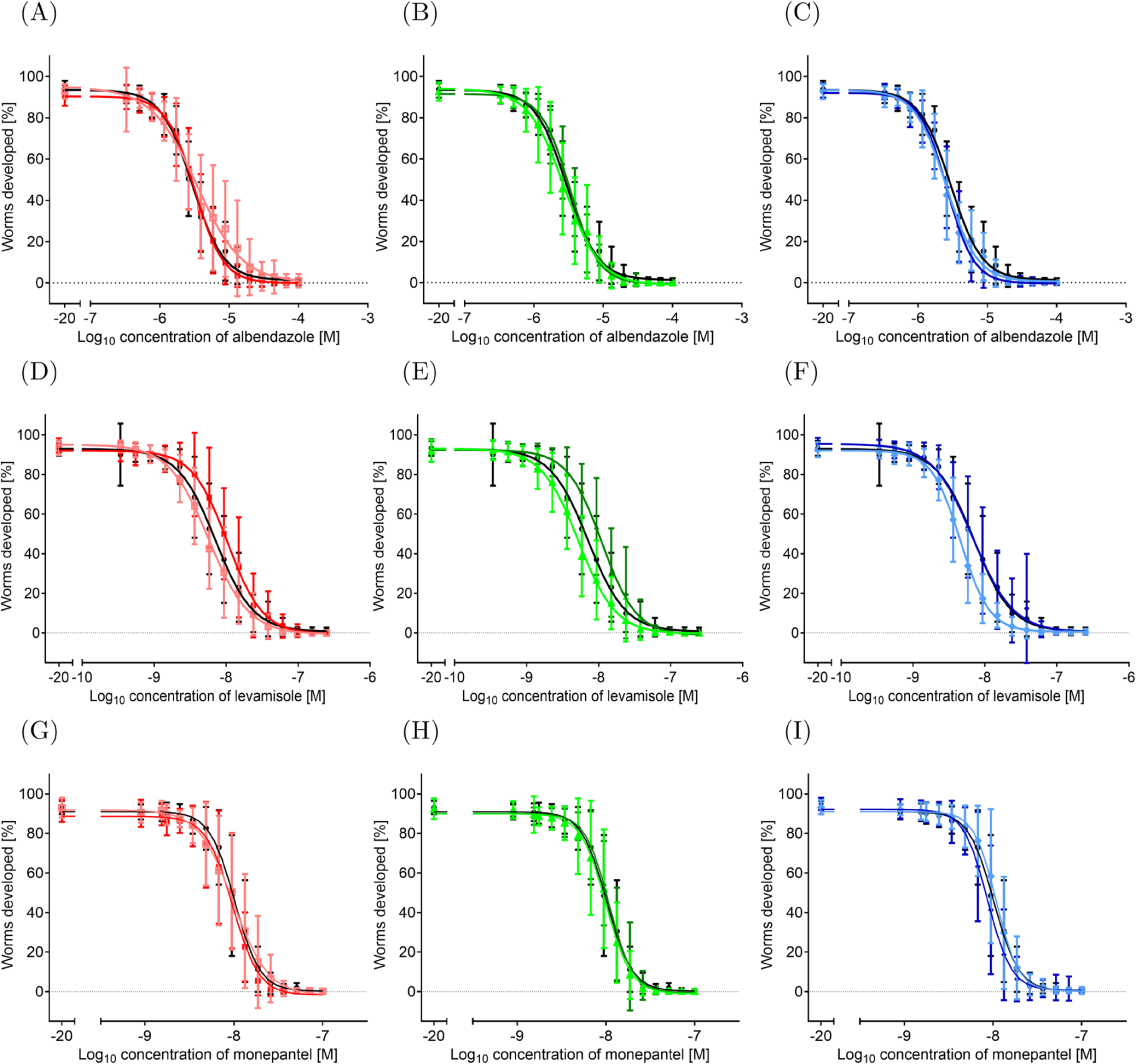
Concentration response curves profiling the responses of the ancestor and the endpoint of the IVM‐treated and control lines in an in vitro larval development assay. Ancestor is a black square, the 2000 IVM‐treated line (2000R) are shown as red squares, the 2000 control line (2000C) as salmon squares, the 1000 IVM‐treated line (1000R) as dark green triangles, the 1000 control line (1000C) as light green triangles, the 200 IVM‐treated line as dark blue diamonds, and the 200 control line as light blue diamonds. (A–C) ABZ, (D–F) LEV, (G–I) MON. Plotted are the mean (solid lines) and standard deviation (error bars) of the 12 population replicates for each worm line. One biological and three technical replicates were done for each population replicate.

The ancestor and the endpoint of the IVM‐treated and control lines were further tested in larval development assays against other classes of anthelmintics: ABZ (benzimidazole), LEV (imidazothiazole), and MON (amino‐acetonitrile). For ABZ, all evolved IVM‐treated lines and control lines showed similar responses, with mean EC_50_ values between 2.54 and 3.41 μM ABZ, to the ancestor (2.95 μM ABZ) (Figure 7A–C, Table 3). In a development assay where the worm length was measured after a 2‐day incubation, 7.5 μM ABZ caused partial growth inhibition (Pallotto et al. 2022). Although most changes in EC_50_ values were significant, but the effect sizes were very small, suggesting that selection for IVM does not lead to systematic changes in susceptibility to ABZ.

Another class of anthelmintics tested was the imidazothiazoles, of which LEV was used. All IVM‐treated and control lines had mean EC_50_ values ranging between 4.58 μM to 10.78 μM LEV (Figure 7D–F). The ancestor had an EC_50_ value of 7.51 μM LEV (Table 4). The 2000 and 1000 IVM‐treated lines significantly increased their mean EC_50_ value compared to the ancestor, but this increase was only 1.4‐fold. The 200 IVM‐treated line had a mean EC_50_ value, which decreased compared to the ancestor's EC_50_ and was not significantly different. All control lines had lower mean EC_50_ values than their corresponding IVM‐treated lines. The mean EC_50_ value of these control lines was also significantly different from the ancestor. Overall, while the 2000 and 1000 IVM‐treated lines showed a significant difference from the ancestor, the fold change was not high enough to be considered resistant. In *C. elegans* , LEV sensitivity is commonly assessed via motility and paralysis assays, with reported effects in the low‐micromolar range, consistent with the concentrations affecting development in our assay (Qian et al. 2008).

**TABLE 4:** EC_50_ values of evolved IVM resistant populations to anthelmintics interacting with nicotinic acetylcholine receptors LEV and MON determined by larval development assays.

Anthelmintic	Population	EC_50_ (nM)	95% CI	*R* ^2^	Fold change^a^	*p* ^b^
LEV	Ancestor	7510	697–809	0.906	1.00	
	2000R	10,540	967–1147	0.880	1.40	< 0.0001
	2000S	5910	550–634	0.925	0.78	< 0.0001
	1000R	10,780	1012–1149	0.920	1.43	< 0.0001
	1000S	5410	506–579	0.925	0.72	< 0.0001
	200R	6950	635–760	0.878	0.92	0.192
	200S	4580	437–479	0.950	0.60	< 0.0001
MON	Ancestor	10.21	9.76–10.68	0.912	1.00	
	2000R	9.64	9.16–10.15	0.894	0.94	0.987
	2000S	9.83	9.14–10.57	0.861	0.96	0.366
	1000R	10.76	10.34–11.20	0.924	1.05	0.084
	1000S	9.98	9.50–10.49	0.895	0.97	0.506
	200R	8.68	8.28–9.10	0.897	0.85	< 0.0001
	200S	10.89	10.42–11.38	0.901	1.06	0.045

^a^ Calculated as EC_50_ resistant or control populations/EC_50_ ancestor.

^b^ Sum of squares *F* test comparing resistant or control populations to the ancestor.

The last anthelmintic tested was MON from the class of amino‐acetonitrile derivatives. The ancestor had an EC_50_ of 10.21 nM MON (Table 4). The IVM‐treated and control lines responded similarly to the ancestor, with the mean EC_50_ values ranging between 8.68 to 10.89 nM MON (Table 4). No drug response pattern was detectable for the lines (Figure 7G–I). The lines that showed a significant difference from the ancestor were the 200 IVM‐treated line and its corresponding control line. The 200 IVM‐treated line had a lower mean EC_50_ than the ancestor, and the 200 control line had a mean EC_50_ value higher than the ancestor. However, as the fold change was around one, no indication of sensitivity or resistance was observed in these populations. Low‐nanomolar concentrations of MON have previously been shown to disrupt developmental progression in *C. elegans* (Rufener et al. 2013).

### 3.6 Lack of a Fitness Cost for IVM Selected Populations

**FIGURE 8:**
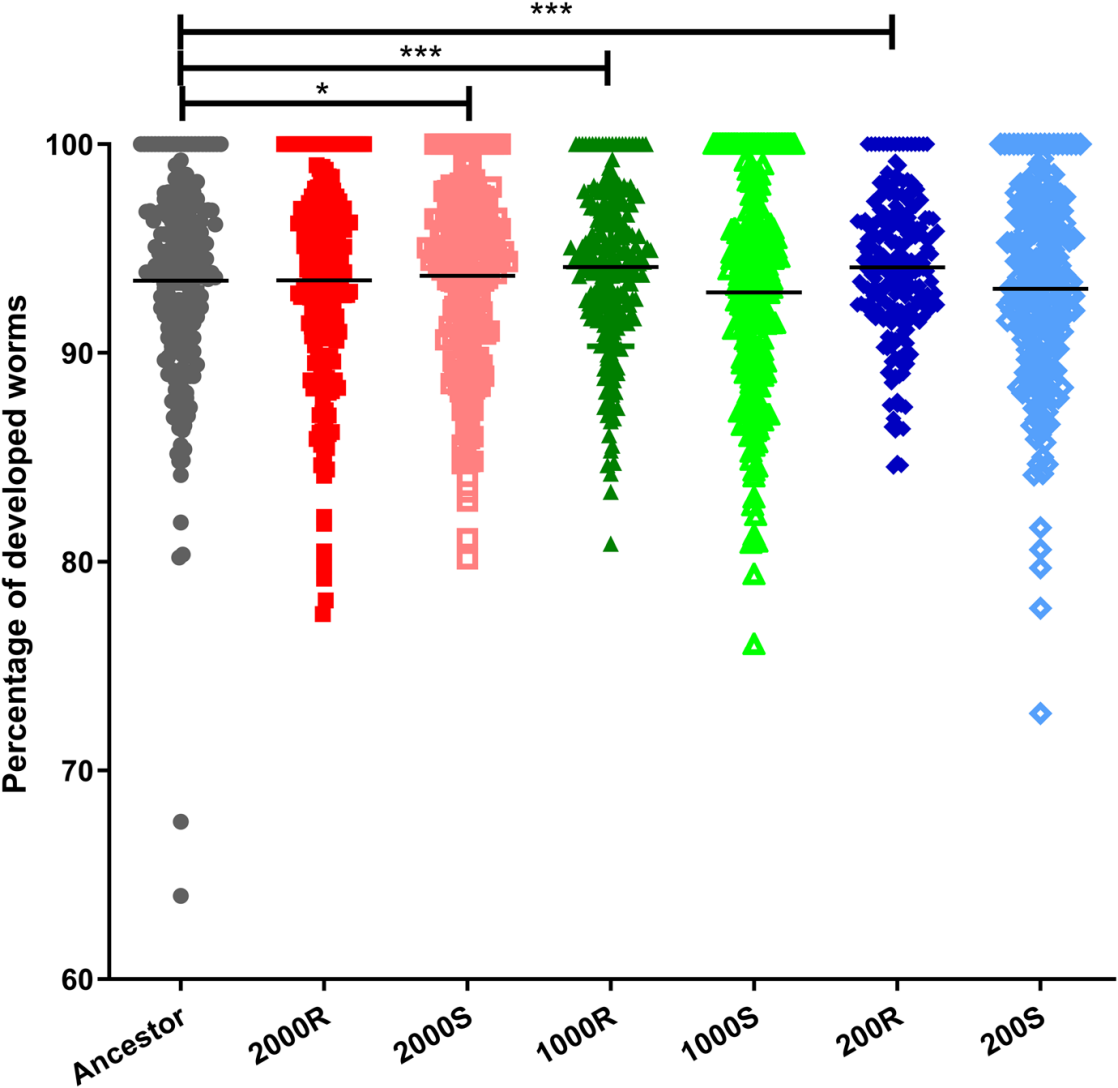
Development of the ancestor and final evolved populations in vehicle controls. Worms were measured for development after a 48 h incubation at 20°C in V medium with 0.4% DMSO. Ancestor is shown as black squares, the 2000 IVM‐treated line (2000R) as red squares, the 2000 control line (2000C) as salmon squares, the 1000 IVM‐treated line (1000R) as dark green triangles, the 1000 control line (1000C) as light green triangles, the 200 IVM‐treated line as dark blue diamonds, the 200 control line as light blue diamonds. The percentage of worms that are fully developed to the L4/adult stage for individual replicates is graphed as a scatter plot. Median is shown. Kruskal‐Wallis test was performed, and no significant differences were found. *p < 0.05; ***p < 0.0001.

The evolution of resistance against specific stressors, including drugs, often comes with a significant fitness cost, especially in the absence of the stressor. Therefore, we assessed whether the evolved populations showed indications of a fitness cost using the larval developmental assay. Worms were allowed to develop in V medium for 48–52 h for the larval development assay (Figure 8). Using the non‐parametric Kruskal–Wallis test, we observed no statistical difference between the lines. No fitness cost was observed for our evolved IVM‐treated lines. Although the distribution of developed worms for the ancestor had a wider range of development than for the evolved populations, further studies and more sensitive testing are needed to determine if there is indeed no fitness cost associated with IVM resistance.

## 4 Discussion

The findings presented in this study shed light on the intrinsic factor of population size and its influence on the rate of IVM resistance evolution in an evolution experiment. While the effect of population size on the rate of drug resistance evolution has been already suggested by the theoretical studies (Trubenová et al. 2025), to our knowledge, this is the first experimental study verifying these claims. In addition to the importance of population size, our study also revealed that males were more frequently observed in the stressed, IVM‐treated lines. Moreover, the IVM‐treated lines appear to produce more mutations than the control lines. These findings from the in vitro evolution experiment presented here were further supported by computational modeling, which successfully mirrored the experimental results and provided insights into the potential outcomes of allowing the worms to evolve for an extended period.

We found that population size strongly and significantly affects how quickly a worm population can evolve resistance to IVM. The 2000 IVM‐treated line adapted the fastest, reaching a final concentration of 15 nM IVM. In contrast, the 200 IVM‐treated line took the longest time to adapt, reaching resistance to only 8 nM IVM (Figure 2A). This 10‐fold difference in size resulted in worms being approximately half as adapted. The required longer adaptation time to reach resistance to 8 nM IVM could also be due to genetic drift as the relative frequency of different genotypes in the 200 population should be much smaller than that in the larger populations.

Although we worked with a derivative of the laboratory wild‐type N2 and not a natural isolate, we included males within the population. In an N2 background, males are usually lost within a few generations under standard experimental evolution conditions (Chasnov and Chow 2002; Cutter et al. 2003; Stewart and Phillips 2002; Wegewitz et al. 2008). Instead, in our evolution experiment, males were consistently maintained at approximately 20% in the presence of IVM and thus at significantly higher levels than under control conditions (Figure 2B,C). Previous work demonstrated that males are favored in *C. elegans* populations in the presence of environmental stressors (Chasnov 2013). For example, males were favored during coevolution of *C. elegans* with bacterial pathogens such as *Serratia marcescens* (Morran et al. 2009) or *Bacillus thuringiensis* (Masri et al. 2013), most likely because of a selective advantage of outcrossing under these conditions (Masri et al. 2013). A similar higher proportion of males was observed in *C. elegans* populations adapting to hyperosmolarity, heat, or chronic irradiation (Piloto et al. 2022; Quevarec et al. 2022; Teotonio et al. 2012). Our observations of the male percentage during the evolution experiment is consistent with past findings. We thus conclude that the outcrossing and, as such, the continuous presence of males similarly provided a selective advantage during experimental adaptation to IVM (Figure 2). Intriguingly, the proportion of males was higher in the more rapidly adapting larger populations, but this could be an artifact due to a stochastic loss of males in the L4/adult stage in the 200 IVM‐treated line. The higher proportion of males in the larger IVM‐treated lines may suggest that the high population size either favors the selection of traits that ensure higher male frequencies or prevents the random loss of males, in both cases promoting the faster adaptation to the environmental stressor.

Assessment by larval development assays showed that all 12 population replicates across the three IVM‐treated lines had similar EC_50_ values (Figure 6, Table 1). Differences in IVM resistance were observed among population size treatments, and the fold change EC50 between IVM‐treated and control lines decreased with decreasing population size. This pattern is similar to a previous study in which IVM resistance was introduced stepwise in a single worm population, resulting in two resistant populations IVR6 that was resistant to 6.9 nM IVM (EC_50_ value = 9.0 nM in larval development assays), and IVR10 resistant to 11.4 nM IVM (EC_50_ value = 36.5 nM) James and Davey (2009). In that study, EC_50_ values substantially exceeded the final selection concentrations, whereas in our experiment EC_50_ values were more closely aligned with the final IVM concentrations used during selection. Thus, although both studies show increasing EC_50_ values with increasing selection intensity, the magnitude of the shift relative to the final selection concentration differed between experiments. Despite differences in experimental design and selection protocols, both studies show a consistent trend in which populations selected to higher IVM concentrations exhibit higher EC50 values in phenotypic assays (James and Davey 2009; Ménez et al. 2016). In addition, the IVM‐treated linesdescribed by James and Davey (2009) were also resistant to MOX, with a 1.5 fold increase in EC_50_ in IVR6 population and a 35 fold increase in EC_50_ in IVR10.

The IVM‐treated lines from this study were also found to have reduced drug efficacy to EMO in addition to reduced drug efficacy to MOX. The observed cross‐drug reduced efficacy to MOX was expected, as both IVM and MOX affect GluCls, causing inhibition, leading to paralysis and death (Dent et al. 2000; Wolstenholme and Rogers 2005). However, the encountered reduced drug efficacy to EMO was not anticipated. EMO activates SLO‐1 (BK) potassium channels and G‐protein‐coupled receptors (GPCRs) in nematodes, leading to paralysis and death (Welz et al. 2011). Mutation in SLO‐1 in *C. elegans* results in worms able to move and reproduce on an EMO concentration of 1 μM. The reduction in EMO efficacy observed here (approximately fivefold) was substantially smaller than that reported for mutations in SLO‐1 (approximately 100‐fold). This difference suggests that the reduced efficacy of EMO is more consistent with changes in the worm's detoxification and stress response pathways. EMO has cleared multi‐resistant *H. contortus* in sheep (Samson‐Himmelstjerna et al. 2005) and *Ancylostoma caninum* in greyhounds (Castro et al. 2020; Samson‐Himmelstjerna et al. 2005). Its anthelmintic effects target sites distinct from other common drugs. Against *Trichuris trichiura*, EMO achieved an 83% cure rate at 5 mg and 100% at 15 mg, compared to 76% for 400 mg of ABZ (Mrimi et al. 2023). Its selectivity and efficacy make EMO a candidate adulticide for human onchocercosis, trichuriasis, and hookworm (Krücken et al. 2021; Mrimi et al. 2023). IVM has been the only drug for onchocercosis control in Africa since the late 1980s, the Americas since the 2000s, and Yemen since 1992 (Al‐Qubati 1994; Dadzie et al. 1991; Rodríguez‐Pérez et al. 2013). While IVM led to many areas eliminating onchocerciasis as a public health problem (Basáñez et al. 2008; Coffeng et al. 2013, 2014; Ozoh et al. 2011; Turner et al. 2014), it mainly targets microfilariae and sterilizes females for only several months. Thus, elimination programs require decades of once‐ or twice‐yearly treatment. Sole reliance on IVM has raised concerns about resistance and reduced sensitivity (Doyle et al. 2017). EMO and other anthelmintics are now considered as additional adulticidal treatments alongside IVM for onchocerciasis control. However, to the best of our knowledge, our study is the first to report that IVM‐resistant *C. elegans* also have reduced drug efficacy to EMO. This finding also raises the question of whether reduced EMO drug efficacy may already be present in IVM‐resistant parasitic nematodes. We suggest that further research should be conducted to assess how widespread similar levels of reduced drug efficacy of ML‐resistant parasitic nematodes to EMO can be found. The findings mentioned above for ML‐resistant *H. contortus* and *A. caninum* suggest that the problem might not be relevant immediately in veterinary practice. Still, the results presented here suggest that close monitoring is advised.

Despite the reduced drug efficacy to EMO, our IVM‐resistant populations showed the same trend as observed by James and Davey in relation to the other classes of anthelmintics (James and Davey 2009). Our IVM‐resistant populations were susceptible to ABZ, similar to their findings. Additionally, there was a small increase in the EC_50_ value for our IVM‐resistant populations to LEV; however, this small change in the EC_50_ value does not indicate the same extent of resistance as seen with MOX or EMO. Furthermore, we tested MON and found that our IVM‐resistant populations remained fully susceptible to this anthelmintic. Although all drugs tested in the larval development assays affect the development of the worms, further phenotypic studies into the specific effects of each anthelmintic on motility, morphology, and reproductive capacity are necessary to fully characterize our IVM‐resistant populations.

Our in vitro evolution experiment was complemented by polygenic computational modeling using a pharmacodynamic framework. In agreement with experimental observations, the model showed that the largest population had the highest rate of resistance evolution under IVM, with quantitative agreement between the concentrations reached by simulated populations and those reached by the in vitro IVM‐treated lines. However, the computational and in vitro methods diverged: the computational model showed that some populations failed to adapt beyond 2.0 nM IVM, which was not observed in the in vitro experiment. It is assumed that, by chance, the populations in the in vitro experiment happened to adapt beyond 2.0 nM IVM. Additionally, if the number of replicates in the in vitro experiment matched those in the model, it is likely that some populations would also fail to adapt beyond 2.0 nM IVM. In this context, it might be an important difference that in the in vitro experiment all replicates were handled in parallel and only exposed to higher IVM concentrations when all replicates were considered to be adapted to the current IVM concentration. If one of the replicates had not evolved resistance above a certain level, this would have stopped further evolution of all replicates. For in silico modeling, all replicates were handled individually.

In the extended model simulating 80 generations, the largest population (2000) remained adapted to a similar concentration of IVM as in the 40 generation model (Figures S6 and S14), while the 1000 and 200 population sizes reached higher concentrations of IVM adaptation in the extended model. This suggests that the initial adaptation in the larger population sizes is more likely due to the increased mutation supply. Larger population sizes have been previously linked to faster rates of resistance in evolution experiments and to genetic diversity (Lanfear et al. 2014; Papkou et al. 2021). However, the computational model was set to mimic the in vitro experiment and not further investigate the extreme effects of population size. Therefore, the difference in population size might not be significant enough to elicit the same response to IVM resistance over long‐term adaptation rates. However, the agreement of the experimental and simulation data suggests that the model can be optimized in the future to predict the risk of resistance evolution in different settings using actual management parameters on farms.

The advantage of the computational model is the ability to generate many repeats and to investigate the evolutionary dynamics and genetic composition in more detail and under a larger parameter space. Our analysis revealed that population size significantly influenced which resistance mutations became fixed in a population. Smaller populations show higher randomness in gene fixation, while larger populations favor mutations with greater benefits. This suggests that the random occurrence of mutations significantly impacts adaptation trajectories, especially in smaller populations (Lanfear et al. 2014; Lenski 2017; Manning and Thompson 1984). In larger populations, subtle differences in the benefits and costs of individual mutations can substantially influence their likelihood of becoming fixed. To thoroughly investigate the fixation of mutations, the computational model was extended from 40 generations to 80. Also, even after 80 generations, none of the populations achieved homozygous mutations on all six loci (Figure 3C,D), suggesting that there is additional potential for resistance evolution.

However, the model may differ from biological reality: while the costs and benefits of individual mutations accumulate, some mutations may epistatically interact in an organism. The model's assumption of multiplicative fitness costs (see Supporting Information for details) might need to be revisited, especially their relevance under continuous IVM exposure. More experimental studies that determine the phenotypic effects of combinations of multiple resistance mutations are needed before the model can be adjusted properly. Previous population genetic modeling studies, for instance, Coffeng et al. (2024) and Schwab et al. (2006), did not assume fitness costs associated with resistance mutations. However, there is insufficient data on the cost of resistance in parasitic worms at the moment. Moreover, how these would combine for mutations at multiple loci is unclear. Obviously, more experimental and modeling research is needed in this area.

The use of *C. elegans* to study the evolution of anthelmintic resistance supports research in parasitic nematodes by providing a model system to identify resistance mechanisms. *C. elegans* has already proven effective in identifying the drug targets of both benzimidazoles and emodepside and in informing the detection of resistance‐associated mutations in parasitic species. In this study, we use *C. elegans* to test how population size influences the rate of resistance evolution. Therefore, its suitability for genetic manipulation and mechanistic analysis highlights the value of this model for studying anthelmintic resistance in parasites. Although *C. elegans* has a shorter life cycle, does not obtain nutrients from a host, and shows a more complex mating system than most parasitic species, it remains a useful and important first step in investigating anthelmintic resistance. Despite these biological differences, the genetic tractability and experimental accessibility of *C. elegans* allow detailed analysis of resistance mechanisms under controlled conditions. To confirm the relevance of these findings, the evolution experiment should be repeated at a smaller scale in parasitic nematode species. From this study, we hope that further work on the effects of population size can be carried out in different parasites to support these conclusions.

## 5 Conclusions

Our study demonstrated that population size impacted the rate of IVM resistance evolution in *C. elegans* and that the frequency of outcrossing was important for rapid adaptation to higher IVM concentrations. The IVM‐treated lines had resistance to MOX and unexpectedly reduced efficacy to EMO, suggesting that experimental evolution favored broad‐range resistance mechanisms. It remains to be clarified if susceptibility to EMO might be reduced in some IVM resistant parasitic nematode populations. Furthermore, the computational model, which was set up using key parameters for the in vitro evolution experiment, showed very similar results, showing that population size is a crucial parameter. Additional modeling also suggested that the effects of population size might level off to some extent in the long term, which indicates that population size is more important for the speed of resistance evolution rather than for the outcome after a high enough number of generations. The additional insight provided by the model suggests that while limited mutational supply plays a critical role in smaller populations, in larger populations, clonal interference leads to the preferential fixation of mutations with larger benefits and smaller fitness costs.

## Funding

Support for this research was funded in part by the Swiss National Science Foundation (SNSF) TMSGI3_218475 to B.T., Volkswagen Foundation (grant numbers 96693 to R.R. and 96695 to G.v.S.H., J.K., H.S.), the Collaborative Research Center (CRC) 1182 on the Origin and Function of Metaorganisms (Project‐ID 261376515m sub‐project A1.1) to H.S., the Max‐Planck Society to H.S., and internal funds of the Institute for Parasitology and Tropical Veterinary Medicine, Freie Universität Berlin to J.H.

## Conflicts of Interest

The authors declare no conflicts of interest.

## Supporting information


**Figure S1:** Assessment of progeny production of male induced *Caenorhabditis elegans* WMB1133 strain population after treatment with ethylmethanesulfonate (EMS). 15 hermaphrodites from untreated and EMS treated (F1 generation) were allowed to lay eggs for 16 h with 1 worm on each NGM plate. The eggs then were incubated for 96 h at 16°C and the number of alive worms on each plate were counted. The average is graphed and shown is the average with the top and bottom bar indicating the standard deviation. A *p*‐value of 0.1668 between the groups was calculcated using a paired *t*‐test in GraphPad Prism (v. 10.5.0).
**Figure S2:**. Concentrations of IVM that were used of the in vitro experiment for the three population lines.
**Figure S3:**. Worm growth during the in vitro evolutionary experiment in comparison to the starting population size. Dashed line indicates the starting population size, the dotted line indicates the population size 10× higher than the starting population size. Pink lines indicate when the population was transferred to a higher concentration of IVM. (A) 2000 population, (B) 1000 population, (C) 200 population.
**Figure S4:** Pharmcodynamic curves of individual strains. Pharmacodynamic curves of various sub‐populations as a function of genotype and ivermectin concentration. The highest fitness of 1 belongs to the wild type in the absence of drugs, and EC50 of the wild type is set to 1. Benefit and cost values are shown in Table S3.
**Figure S5:** Fitness of individual strains. The fitness of individual genotypes at ivermectin concentrations 0, 2, 10 and 15 nM. Different colors represent the number of loci that carry two mutated alleles.
**Figure S6:** Distribution of the final concentration reached after 4 0 generations. Horizontal lines represent medians.
**Figure S7:** Genotype frequencies of 15 randomly selected final populations of 200 individuals, after 40 generations of evolution. On the *X*‐axis, different loci are portrayed, and on the *Y*‐axis, the genotypes that are possible for each locus. The rectangle's color corresponds to the fraction of individuals having a particular genotype on that particular locus. Values in each column add to 1.
**Figure S8:** Genotype frequencies of 15 randomly selected final populations of 1000 individuals, after 40 generations of evolution. On the *X*‐axis, different loci are portrayed, and on the *Y*‐axis, the genotypes that are possible for each locus. The rectangle's color corresponds to the fraction of individuals having a particular genotype on that particular locus. Values in each column add to 1.
**Figure S9:** Genotype frequencies of 15 randomly selected final populations of 2000 individuals, after 40 generations of evolution. On the *X*‐axis, different loci are portrayed, and on the *Y*‐axis, the genotypes that are possible for each locus. The rectangle's color corresponds to the fraction of individuals having a particular genotype on that particular locus. Values in each column add to 1.
**Figure S10:** Male fraction over time. The solid line represents the mean of 100 stochastic simulations, while the shading represents the standard deviation.
**Figure S11:** Concentration at which the worm population grew as a function of time. Blue solid lines correspond to individual simulations; the red dashed line represents the mean.
**Figure S12:**. Distribution of the final concentration reached after 80 generations. Horizontal lines represent medians.
**Figure S13:** Simulations of the neutral evolution. The average number of homozygous mutations per worm present within the population at a certain time point: 200 (blue), 1000 (green) and 2000 (red).
**Figure S14:** The average genotype frequencies of 100 final populations. Different population sizes: (a) 200, (b) 1000, (c) 2000. On the *X*‐axis, different loci are portrayed, and on the *Y*‐axis, the genotypes that are possible for each locus. The rectangle's color corresponds to the fraction of individuals having a particular genotype on that particular locus. Values in each column add to 1.

**Table S1:** Genomic mutation rate of evolved populations during the evolution experiment.
**Table S2:**. Increase resistance of *C. elegans* against ivermectin.
**Table S3:** Mutational effects used in the simulations.
**Table S4:** Sequence of ivermectin concentrations in nM.

## Data Availability

All in vitro data supporting the findings of this study are included in the manuscript and Supporting Information. The in silico computational model data is available at https://github.com/Trubenova/EvolutionOfIvermectinResistance.
