# Supplementary material for: Evolution of Ivermectin Resistance in the Nematode Model Caenorhabditis elegans : Critical Influence of Population Size and Altered Emodepside Efficacy
Source: Evol Appl. 2026 Apr 24;19(4):e70241. doi: 10.1111/eva.70241 (PMC13108422; doi:10.1111/eva.70241)
Supplement: Supplementary file 1 — Figure S1: Assessment of progeny production of male induced Caenorhabditis elegans WMB1133 strain population after treatment with ethylmethanesulfonate (EMS). 15 hermaphrodites from untreated and EMS treated (F1 generation) were allowed to lay eggs for 16 h with 1 worm on each NGM plate. The eggs then were incubated for 96 h at 16°C and the number of alive worms on each plate were counted. The average is graphed and shown is the average with the top and bottom bar indicating the standard deviation. A p‐value of 0.1668 between the groups was calculcated using a paired t‐test in GraphPad Prism (v. 10.5.0). Figure S2:. Concentrations of IVM that were used of the in vitro experiment for the three population lines. Figure S3:. Worm growth during the in vitro evolutionary experiment in comparison to the starting population size. Dashed line indicates the starting population size, the dotted line indicates the population size 10× higher than the starting population size. Pink lines indicate when the population was transferred to a higher concentration of IVM. (A) 2000 population, (B) 1000 population, (C) 200 population. Figure S4: Pharmcodynamic curves of individual strains. Pharmacodynamic curves of various sub‐populations as a function of genotype and ivermectin concentration. The highest fitness of 1 belongs to the wild type in the absence of drugs, and EC50 of the wild type is set to 1. Benefit and cost values are shown in Table S3. Figure S5: Fitness of individual strains. The fitness of individual genotypes at ivermectin concentrations 0, 2, 10 and 15 nM. Different colors represent the number of loci that carry two mutated alleles. Figure S6: Distribution of the final concentration reached after 4 0 generations. Horizontal lines represent medians. Figure S7: Genotype frequencies of 15 randomly selected final populations of 200 individuals, after 40 generations of evolution. On the X‐axis, different loci are portrayed, and on the Y‐axis, the genotypes that are possibl [file EVA-19-e70241-s002.pdf]

Supplementary Material

Contents

S1 *In vitro* evolution experiment 2

S2 Computational model 5

S2.1 Model setup . . . 5
S2.2 Simulated scenarios . . . 10
S2.2.1 Simulations of ivermectin resistance evolution . . . 10
S2.2.2 Long-term experiment . . . 10
S2.2.3 Neutral evolution . . . 10
S2.3 Results . . . 11
S2.3.1 Simulations of ivermectin resistance evolution . . . 11
S2.3.2 Long-term experiment . . . 17
S2.3.3 Neutral evolution . . . 18

List of Figures

S1 Assessment of progeny production of male induced *C. elegans* WMB1133 strain population after treatment with Ethylmethanesulfonate (EMS). . . . 2
S2 Concentrations of IVM that were used for the in vitro experiment for the three population lines. 2
S3 Worm growth during the *in vitro* evolutionary experiment in comparison to the starting population size. . . . 3
S4 Pharmcodynamic curves of individual strains. . . . 6
S5 Fitness of individual strains. . . . 7
S6 Distribution of the final concentration reached after 40 generations. . . . 11
S7 Genotype frequencies of 15 randomly selected final populations of 200 individuals, after 40 generations of evolution. . . . 13
S8 Genotype frequencies of 15 randomly selected final populations of 1000 individuals, after 40 generations of evolution. . . . 14
S9 Genotype frequencies of 15 randomly selected final populations of 2000 individuals, after 40 generations of evolution. . . . 15
S10 Male fraction over time. . . . 16
S11 Concentration at which the worm population grew as a function of time. . . . 17
S12 Distribution of the final concentration reached after 80 generations. . . . 17
S13 Simulations of the neutral evolution. . . . 18
S14 The average genotype frequencies of 100 final populations . . . 18

List of Tables

S1 Genomic mutation rate of evolved populations during the evolution experiment. . . . 4
S2 Increase resistance of *C. elegans* against ivermectin. . . . 9
S3 Mutational effects used in the simulations . . . 10
S4 Sequence of ivermectin concentrations in nM. . . . 10

S1 *In vitro* evolution experiment

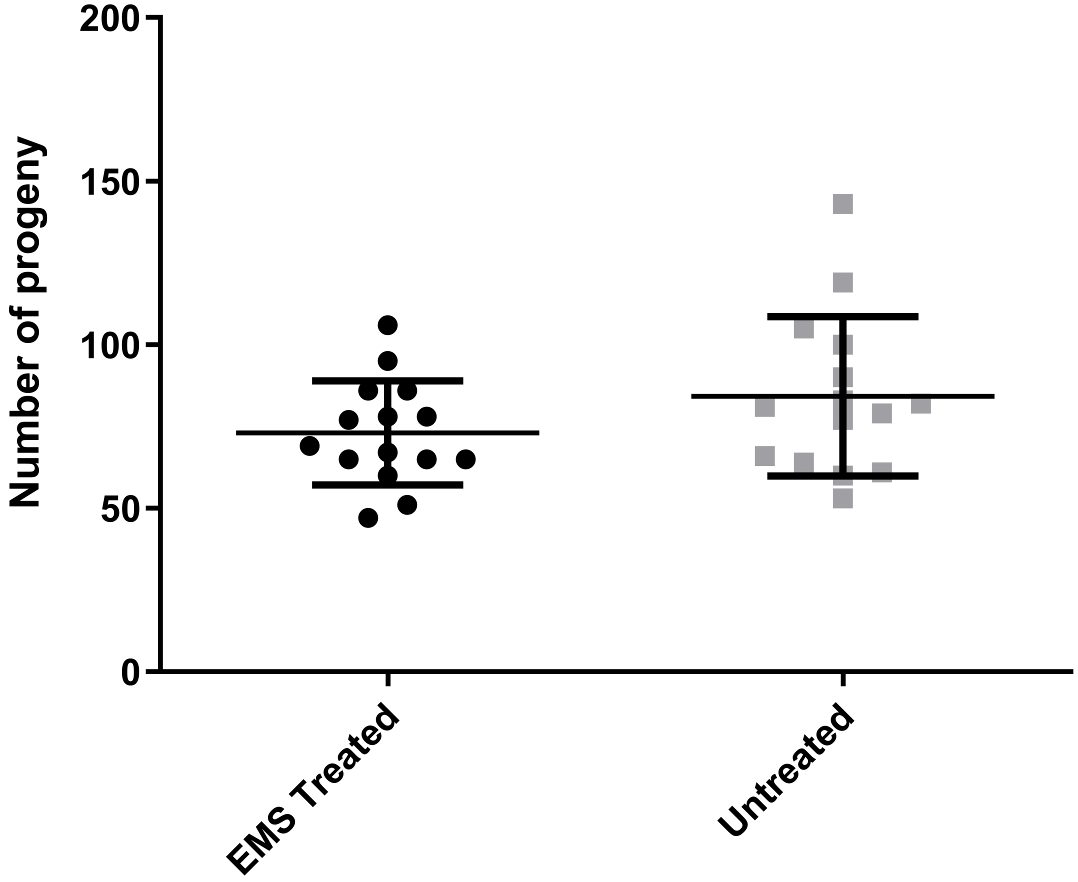

Figure S1: **Assessment of progeny production of male induced *C. elegans* WMB1133 strain population after treatment with Ethylmethanesulfonate (EMS).**
Hermaphrodites (n = 15) from untreated and EMS treated (F1 generation) were allowed to lay eggs for 16 hours with 1 worm on each NGM plate. The eggs then were incubated for 96 hours at 16 deg$C$ and the number of alive worms on each plate were counted. The average is graphed and the average ± standard deviation are plotted.

2000- and 1000-IVM treated lines

0.1	0.2	0.4	0.8	1.0	1.5	2.0	2.5	3.0	4.0	5.0	6.0	8.0	10.0	12.0	15.0*

200-IVM treated line

0.1	0.2	0.4	0.8	1.0	1.5	2.0	2.5	3.0	3.5	4.0	4.5	5.0	6.0	8.0

Figure S2: **Concentrations of IVM that were used for the in vitro experiment for the three population lines.**

(A)

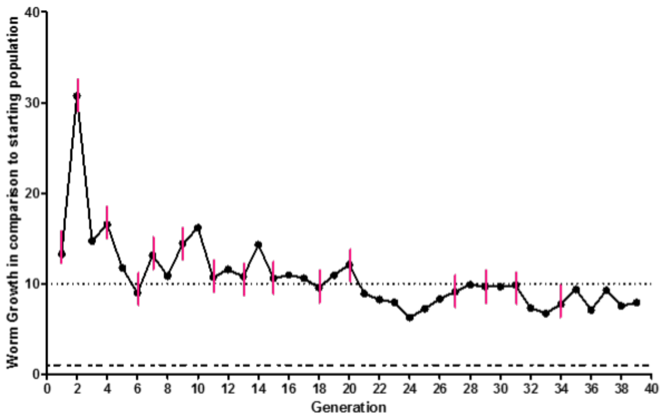

(B)

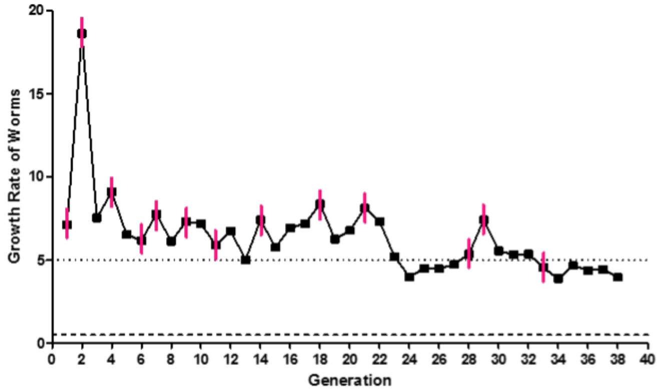

(C)

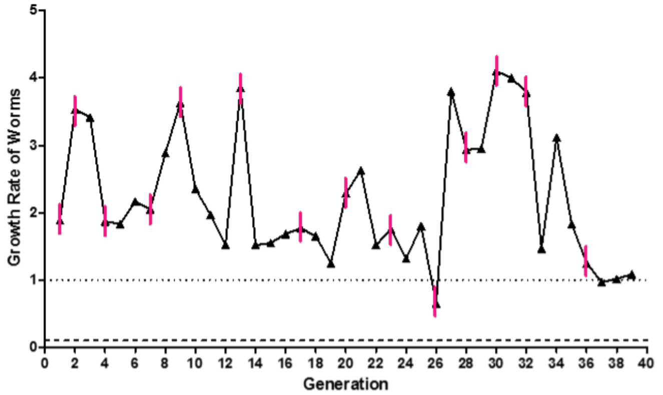

Figure S3: **Worm growth during the *in vitro* evolutionary experiment in comparison to the starting population size.**
Dashed line indicates the starting population size, the dotted line indicates the population size 10X higher than the starting population size. Pink lines indicate when the population was transferred to a higher concentration of IVM. A) 2000 population, B) 1000 population, C) 200 population.

Table S1: Genomic mutation rate of evolved populations during the evolution experiment.

**Population**	**Total number of *de novo* mutations**	**Mutation rate (per site per genome)**
2000R	4082	10.46×10^{-7}
2000C	2860	7.33×10^{-7}
1000R	3973	10.18×10^{-7}
1000C	2949	7.56×10^{-7}
200R	2427	6.22×10^{-7}
200C	2447	6.27×10^{-7}

S2 Computational model

The computation model used to simulate the evolutionary experiment is based on the population genetic, compartmental model proposed in [Trubenová et al., 2025] with a few modifications, as explained below.

S2.1 Model setup

Genetics Drug resistance of *C. elegans* to ivermectin involves complex polygenic mechanisms with mutations of varying effects. Therefore, in our model, drug resistance in the worm population is encoded by six independent, unlinked diploid loci. This generates $3^6 = 729$ possible genotypes (also referred to as 'strains'), providing substantial complexity to reflect the diverse resistant strains observed in experimental settings (see Table S2 and reference herein) while remaining computationally tractable. Any genotype can belong to a male or a hermaphrodite, resulting in 1458 different types of worms, sometimes referred to as sub-populations and modelled as independent compartments.

We model the evolution of drug resistance under the assumption that mutations confer both a fitness cost (reduced fitness in drug-free environments) and a benefit (increased EC50 or drug tolerance). We denote the wild-type allele as '0' and the mutated allele as '1'. We assume that all mutations are recessive, with varying effects (Table S3). Therefore, both the wild-type homozygous locus (00) and the heterozygous locus (01 or 10) have a phenotypic value of 0, meaning they will not experience the effects of the mutation. Only the mutated homozygous locus (11) confers both the fitness benefit and the cost associated with the mutation, resulting in a phenotypic value of 1.

Pharmacodynamics Homozygous mutations confer fitness cost to the carrier - they reduce its fitness in the absence of drugs, but increase its tolerance to it (EC50). For each genotype j, we use a vector of phenotypic values $\mathbf{p_j}$ to calculate its total benefit and cost associated with this genotype. Benefits and costs of individual loci are determined by vectors $\mathbf{b}$ and $\mathbf{c}$, respectively. They combine additively to determine the total benefit and cost associated with each genotype as $B_i = \mathbf{p_i} \cdot \mathbf{b} = \sum_j p_{ij} b_j$ and $C_i = \mathbf{p_i} \cdot \mathbf{c} = \sum_j p_{ij} c_j$, respectively.

The total benefit defines the increase of the EC_{50} of this strain as $EC50_i = EC50_{wt} + B_i$, where EC_{50wt} is the EC_{50} of the sensitive wild-type. The maximum fitness of the strain and its $EC50$ value enable us to define its pharmacodynamic curve (Figure S5, A), which, in turn, allows for the calculation of fitness in response to the drug concentration (Figure S5, B-D).

$$\phi_i(A) = (1 - C_i) \left(1 - \frac{\left(\frac{A}{EC50_i} \right)^{\kappa}}{\left(\frac{A}{EC50_i} \right)^{\kappa} + 1} \right) \tag{1}$$

where A is the drug concentration and κ defines the steepness of the curve.

Various anthelmintic drugs affect worms differently depending on their life cycle stage, with effects including killing adult worms, paralyzing them, inhibiting reproduction, or preventing egg hatching (see Trubenová et al. [2025] for details). However, for simplicity, we assume that any reduction in fitness, whether due to mutation or the effect of the drug, manifests as a decreased number of hatched eggs, which affects the offspring production of specific genotypes. All hatched offspring survive to adulthood and can reproduce. To calculate the number of laid and hatched eggs in this study, the fitness of the hermaphrodite was multiplied by 300 when selfing and by 1000 when mating [Kimble and Ward, 1988].

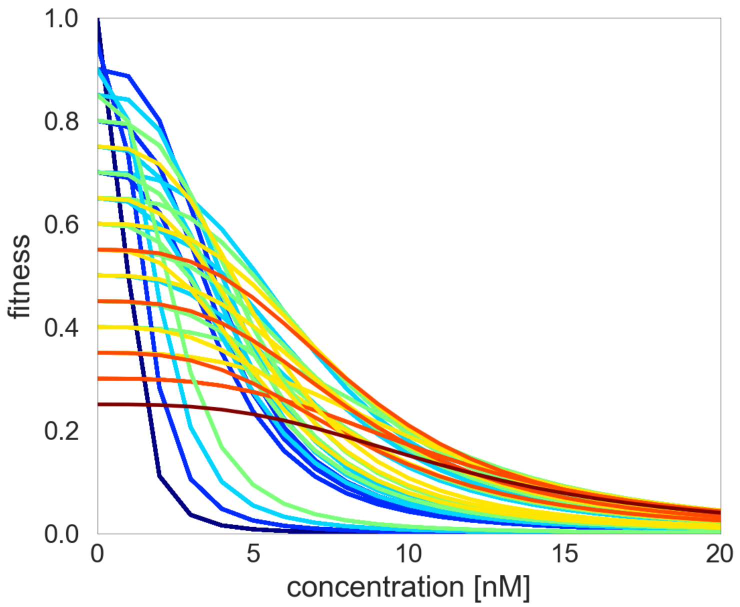

Figure S4: **Pharmcodynamic curves of individual strains.**
Pharmacodynamic curves of various sub-populations as a function of genotype and ivermectin concentration. The highest fitness of 1 belongs to the wild type in the absence of drugs, and EC50 of the wild type is set to 1. Benefit and cost values are shown in Table S3.

Reproduction Following the experimental setup, we assume that the initial male fraction is 20%. The fraction of hermaphrodites undergoing mating is 10%, leading to a decline in the male population to 10 - 15%, which is consistent with experimental observations in this and previous experiments [Wegewitz et al., 2008]. We further assume that hermaphrodites mate with males in proportion to their respective numbers. The genotypes of the offspring are then determined using Punnett squares to calculate the probabilities.

In *C. elegans*, sex is determined by the count of X chromosomes present; individuals with two X chromosomes (XX) are hermaphrodites, while those with just one X chromosome (XO) are males. A male *C. elegans* produces both sperm that carry an X chromosome and sperm that carry no X chromosome (O-carrying). Therefore, half of the offspring generated by outcrossing (see the definition of egg count) in each genotype class are males, while the other half is allocated to be hermaphrodites [Bahrami and Zhang, 2013, Strome et al., 2014].

The remainder of hermaphrodites reproduces through selfing. All sperm produced by hermaphrodites carry an X chromosome, which means that selfing results in all offspring being hermaphrodites [Bahrami and Zhang, 2013, Strome et al., 2014]. However, multiple genotypes arise, in the ratios calculated using Punnett squares (see [Trubenová et al., 2025], Reproduction).

Simulation process To follow the *in vitro* experiment, the starting population of desired size (200, 1000 and 2000 individuals) was seeded in the wild type compartment, split between hermaphrodites and males in a 4:1 ratio (20% male present in the population). The concentration of ivermectin was set to 0. Then, we simulated 40 (80) cycles, corresponding to 40 (80) generations, according to the following procedure:

1. **Mating:** 10% of hermaphrodites mated with males. The number of eggs laid was calculated using pharmacodynamic curves, considering the genotypes of the hermaphrodites and the ivermectin concentration.

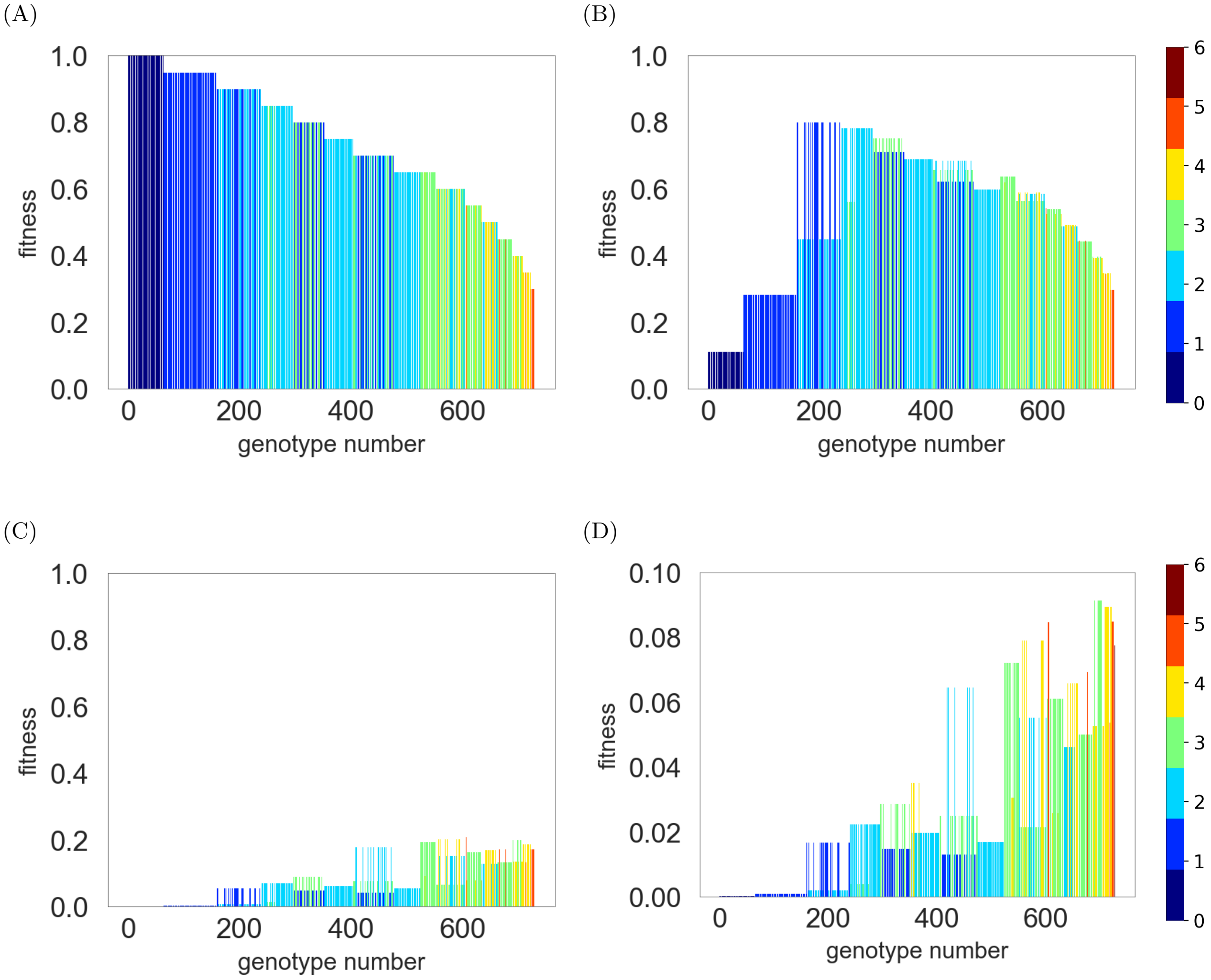

Figure S5: **Fitness of individual strains.**
The fitness of individual genotypes at ivermectin concentrations 0, 2, 10 and 15 nM. Different colours represent the number of loci that carry two mutated alleles.

2. **Offspring Allocation:** New offspring were assigned genotypes based on their parents' pairing (see above). They were then evenly divided between male and hermaphrodite compartments.

3. **Selfing:** Unmated hermaphrodites reproduced by selfing. The number of eggs was determined using pharmacodynamic curves. Offspring were assigned genotypes accordingly and were all considered hermaphrodites.

4. **Mutation:** A fraction of offspring, determined by the mutation rate μ) and population size, were assigned mutated genotypes, redistributing them to different compartments (see Trubenová et al. [2025], Mutation).

5. **Population Replacement:** Offspring replaced the parental population.

6. **Adaptation and Dilution:**

   - If the population increased at least 30-fold, it was considered adapted. The drug concentration increased according to a predefined sequence (Table S4), and the population was diluted to its

original size.

- If the population did not meet the adaptation threshold, the drug concentration remained unchanged, but the population was still diluted to its original size.

Simulations were terminated after 40 (80) generations.

Table S2: **Increase resistance of *C. elegans* against ivermectin.**

gene/strain	value	fold increase	reference
N2	3.693 nM		Janssen et al. [2013]
che-1	lot more than 10 nM		Page [2018]
che-11	lot more than 10 nM		Page [2018]
che-13	lot more than 10 nM		Page [2018]
osm-5	lot more than 10 nM		Page [2018]
daf-10	lot more than 10 nM		Page [2018]
dyf- 3	lot more than 10 nM		Page [2018]
dyf-4	lot more than 10 nM		Page [2018]
dyf-7	lot more than 10 nM		Page [2018]
mec-8	lot more than 10 nM		Page [2018]
bbs-1	lot more than 10 nM		Page [2018]
N2 parental		1	James and Davey [2009]
IVR6 (multiple mutations)		4.5	James and Davey [2009]
IVR10 (multiple mutations)		19	James and Davey [2009]
wild type (multiple mutations)	EC50 = 1.69 nM		Ménez et al. [2016]
IVR10 (multiple mutations)	EC50 = 12.43 nM		Ménez et al. [2016]
normal strain	IC50 = 0.03 μM		Mathew et al. [2016]
DA1316 (several mutations)	IC50 = 7 μM		Mathew et al. [2016]
wt	EC37=1.1 ng/ml		Dent et al. [2000]
avr-15, UNC-7	EC37=733 ng/ml		Dent et al. [2000]
avr-15, avr-14, glc-1	EC37=4264 ng/ml		Dent et al. [2000]
avr-15, avr-14	EC37=13,8 ng/ml		Dent et al. [2000]

S2.2 Simulated scenarios

S2.2.1 Simulations of ivermectin resistance evolution

The first set of simulations was carried out while the *in vitro* experiment was underway. Due to the lack of information regarding EC50 values of possible mutations, we assumed that the EC50 of the starting, sensitive ancestral population was 1 nM.

We considered three loci with small effects and 3 with larger effects on EC_{50}, as shown in Table S3. The benefit values were chosen such that the mutants are consistent with the range of EC50 values found in the literature (see Table S2), while the cost values have been generated arbitrarily, due to the lack of information, assuming that the costs are relatively low. Furthermore, we also assumed that a larger mutational benefit was associated with a higher cost, manifesting as a lower egg count in the absence of the drug. The fitness of different genotypes in various drug concentrations is shown in Figure S5.

Table S3: **Mutational effects used in the simulations**

Locus	1	2	3	4	5	6
Benefit	0.5	0.5	0.5	3	3	3
Cost	0.05	0.05	0.05	0.1	0.2	0.3

The mutation rate was set to $\mu = 10^{-4}$, assuming only one mutation per offspring. This rough estimate is based on the mutation rate in the *C. elegans* per site per generation ($\approx 2.1 \times 10^{-8}$, Denver et al. [2004]), the median size of coding genes in this organism ($\approx 2 \times 10^3$ base pairs, [Spieth et al., 2014]), and the assumptions of six resistance coding genes (yielding 2.4×10^{-4} mutation rate). However, as most mutations are not beneficial, we decreased the mutation rate in our model to $\mu = 10^{-4}$, which is still, very likely, an overestimate.

Simulations were stochastic, repeated 100 times for the same initial conditions. To follow the *in vitro* experiment, the starting population of desired size (200, 1000 and 2000 individuals) was seeded in the wild type compartment, split between hermaphrodites and males in 4:1 ratio (corresponding to 20% male present in the population). The concentration of ivermectin was set to 0. Then, we simulated 40 cycles according to the simulation process described above. The amount by which the drug concentration was increased when the population had adapted was the same as the one used in the *in vitro* experiment, shown in Table S4.

Table S4: **Sequence of ivermectin concentrations in nM.**

0	0.1	0.2	0.4	0.8	1	1.5	2	2.5	3	4	5	6	8	10	12	15	18	21	24	28	32	36	40	45

S2.2.2 Long-term experiment

To simulate a longer-running experiment, we extended the simulation time of the simulations described above to 80 generations, with 50 stochastic simulations performed for each population size. All other experimental conditions were identical to simulations of ivermectin resistance evolution described above.

S2.2.3 Neutral evolution

To establish a baseline for comparison, we performed a control simulation modelling the neutral evolution of populations in a drug-free medium. This simulation maintained all other experimental conditions identical to those used in the simulations of ivermectin resistance evolution.

S2.3 Results

S2.3.1 Simulations of ivermectin resistance evolution

From the time course of individual simulations (Figure 3 A-C), we see that in the initial phase of the experiment (first 10 generations), all populations were able to progress to higher concentration at each generation, which corresponds to reaching a concentration of 2.5 nM. This is caused by the fact that with male presence, the expected number of laid eggs was high enough to satisfy the condition for concentration increase (average egg count over 30) even for sensitive wild type, and no mutations were necessary (see Figure S5 A).

In later generations and higher concentrations, we see that evolutionary trajectories differ between individual simulations. While some did not cross the threshold of 2.5 nM, in some simulation trials, even the small population could adapt to 15 nM within the given time frame of 40 generations (Figure 3 A). This is the consequence of the stochastic nature of the simulations - different mutations arose at different times. As can be expected, individual evolutionary trajectories differ more for small populations, as the stochasticity in mutation appearance plays a more critical role. In large populations, mutation supply was a much less limiting factor, and the trajectories were more similar. Figure S6 and Figure 3 D - F show the distribution of the final concentrations reached for three different population sizes. Figure 4 A shows the mean and standard deviation of the concentration at which the worm population grew as a function of time for all three populations.

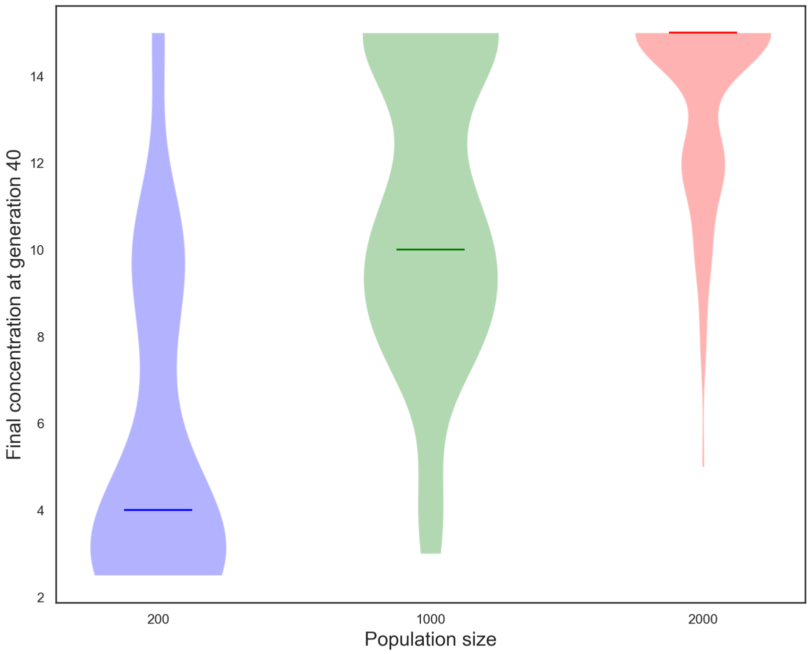

Figure S6: **Distribution of the final concentration reached after 40 generations.** Horizontal lines represent medians.

The final genotype frequencies of the populations at generation 40 differed profoundly among different simulation trials. While the average number of homozygous mutants increased over time in all three sample sizes (Figure 4 B), in none of the simulations were full mutants observed, suggesting that the populations could evolve further.

To get a better insight into the composition of the final population reached after 40 generations, we look at each locus individually. Figures S7 - S9 show the proportion of individuals that were homozygous sensitive (bottom row), heterozygous (thus also sensitive, middle row) and homozygous resistant (top row) at each of the loci (columns) in the final population of 15 randomly selected simulation trials.

These figures show that the genotype frequencies of the final population indeed varies profoundly, as expected. Moreover, we observed that in populations with 200 individuals, any of the loci could become homozygous with resistant alleles without any clear difference between the loci, which manifests as an average value of homozygous resistant mutant around 1/6 in all loci. We hypothesize that the limited mutational supply causes this phenomenon; the first mutation to occur is the most likely to become fixed.

On the other hand, in larger populations of 1000 and 2000 individuals, resistant loci are mostly those of larger effects (locus 4, 5 and 6). This suggests that unlike in the small population, clonal interference, rather than limited mutational supply, hindered adaptation in these larger populations.

The fraction of males is independent of the environment, only given by the willingness of hermaphrodites to mate and their fecundity when selfing and mating (See Equation 5 in Trubenová et al. [2025]), resulting in a stable male fraction of 0.135. Therefore, we observed a quick decline in the fraction of males in all the population sizes in our simulations. As the simulations are stochastic, the fraction of males oscillates around the calculated value.

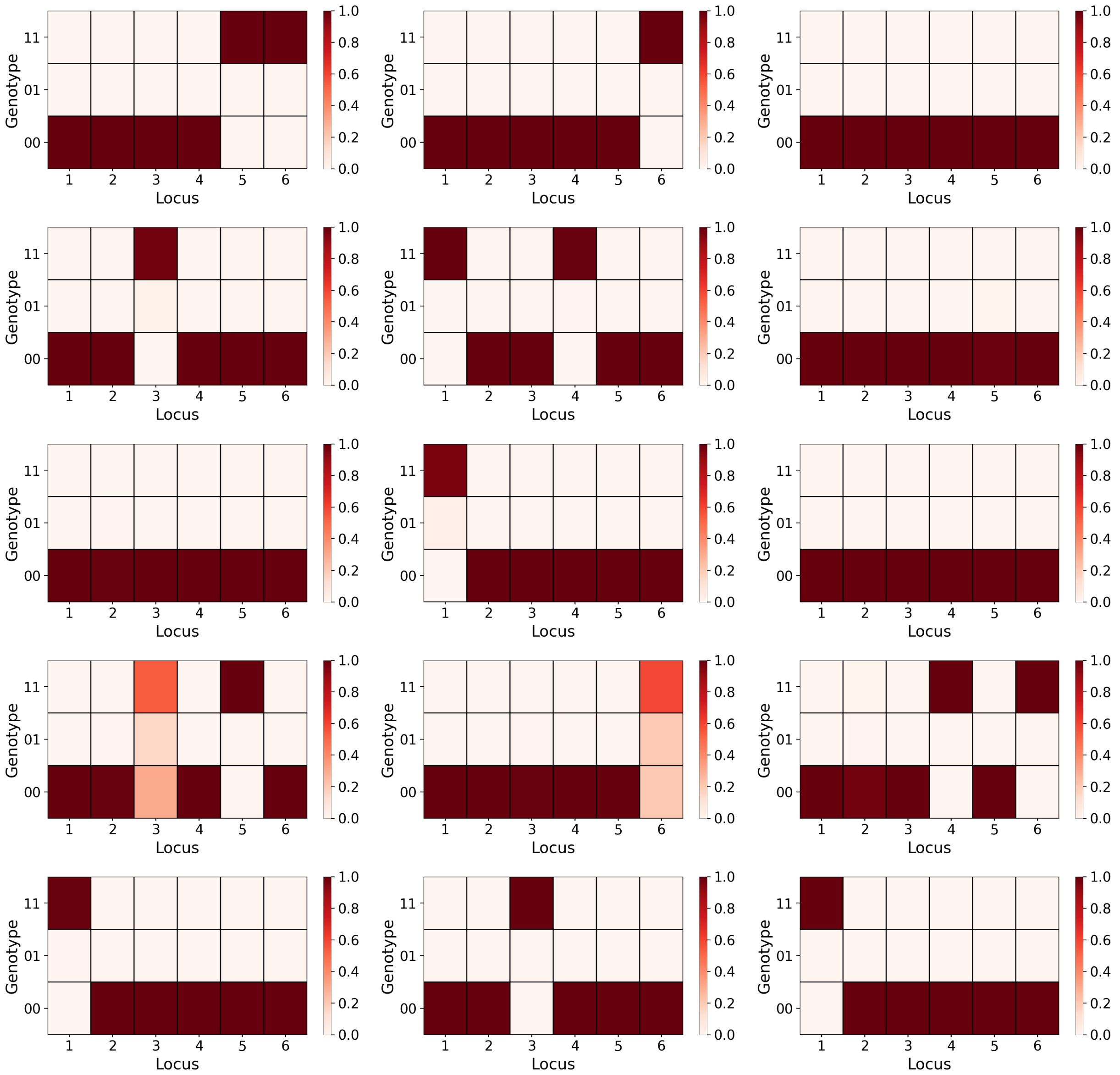

Figure S7: **Genotype frequencies of 15 randomly selected final populations of 200 individuals, after 40 generations of evolution.**
On the X axis, different loci are portrayed, and on the Y axis, the genotypes that are possible for each locus. The rectangle's colour corresponds to the fraction of individuals having a particular genotype on that particular locus. Values in each column add to 1.

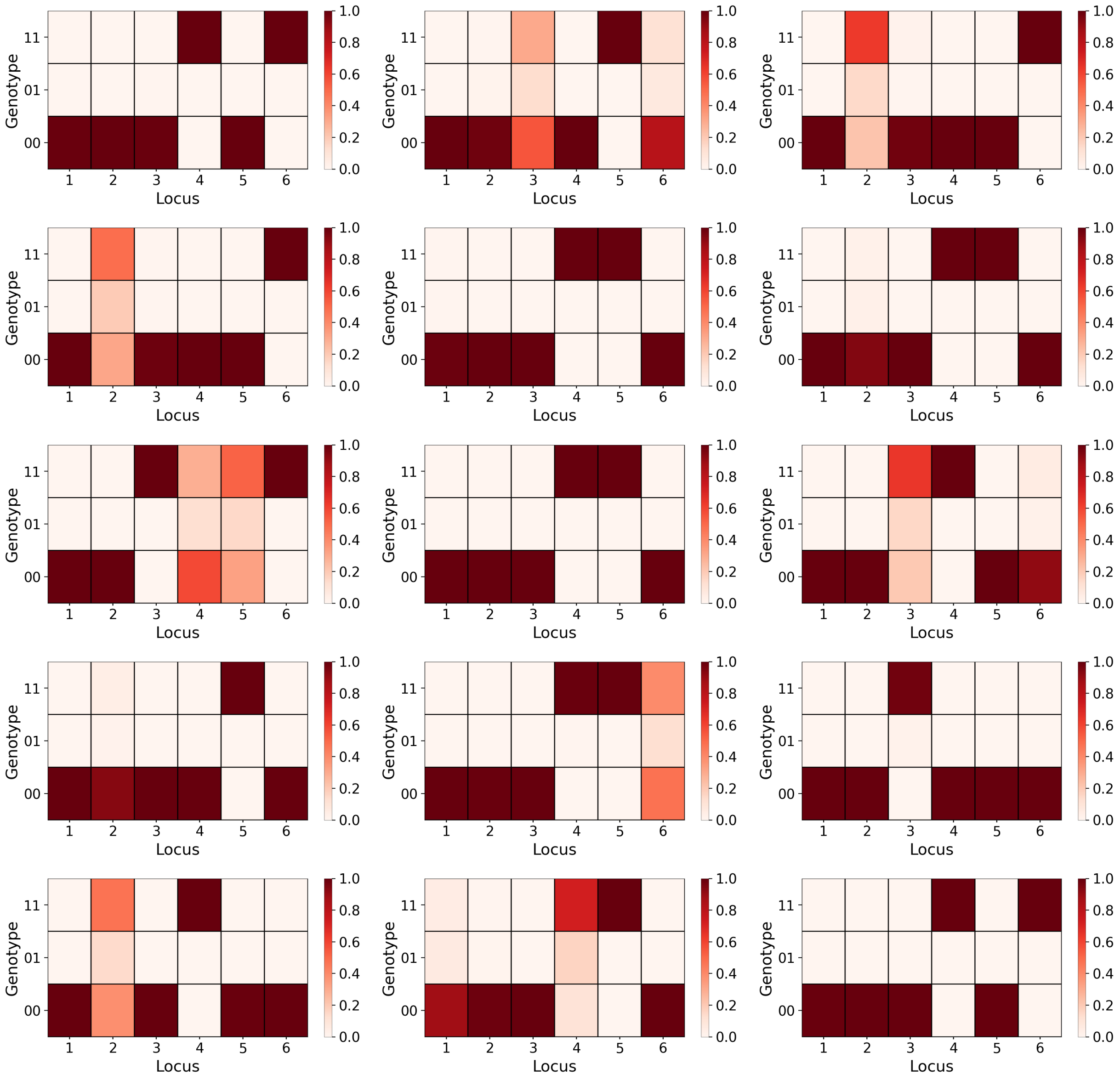

Figure S8: **Genotype frequencies of 15 randomly selected final populations of 1000 individuals, after 40 generations of evolution.**
On the X axis, different loci are portrayed, and on the Y axis, the genotypes that are possible for each locus. The rectangle's colour corresponds to the fraction of individuals having a particular genotype on that particular locus. Values in each column add to 1.

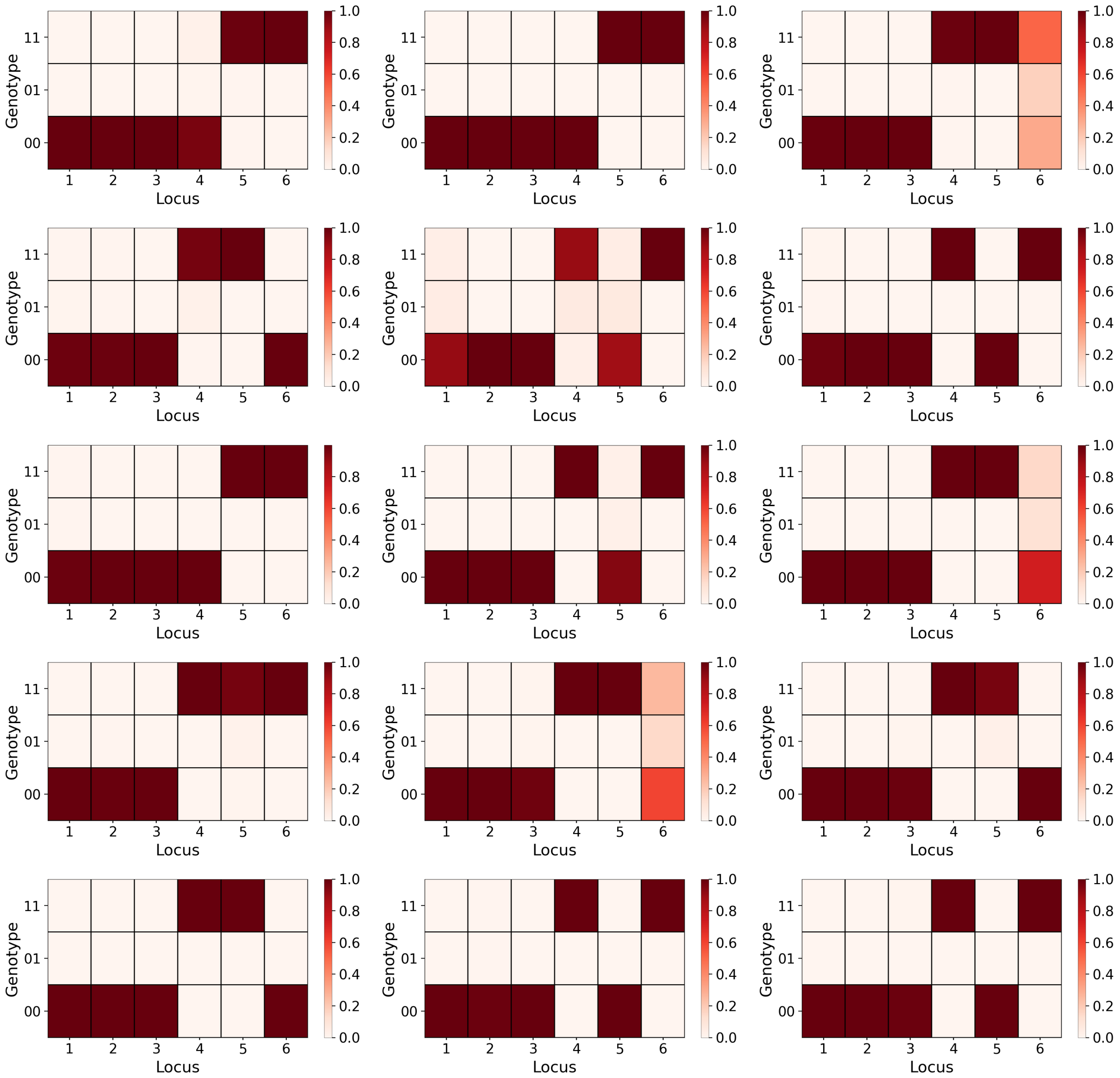

Figure S9: **Genotype frequencies of 15 randomly selected final populations of 2000 individuals, after 40 generations of evolution.**
On the X axis, different loci are portrayed, and on the Y axis, the genotypes that are possible for each locus. The rectangle's colour corresponds to the fraction of individuals having a particular genotype on that particular locus. Values in each column add to 1.

(A) Population size of 200 (B) Population size of 1000 (C) Population size of 2000

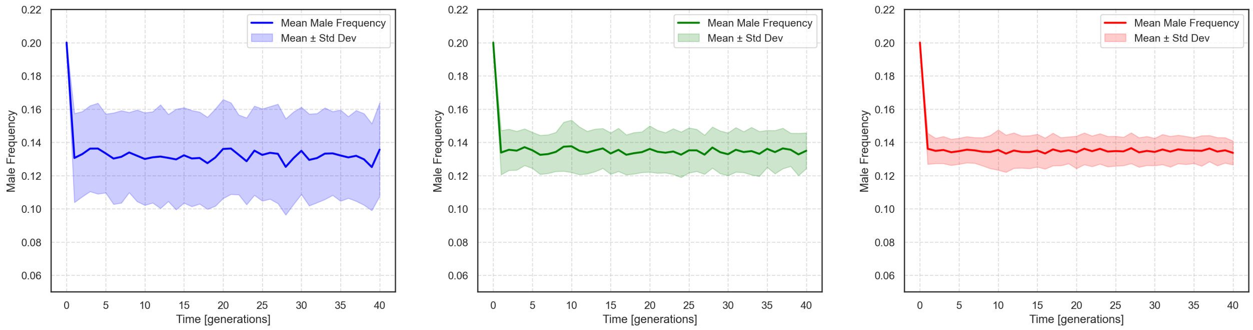

Figure S10: **Male fraction over time.**
The solid line represents the mean of 100 stochastic simulations, while the shading represents the standard deviation.

S2.3.2 Long-term experiment

In the long-term experiment, populations of *C. elegans* were allowed to evolve for 80 generations. The smaller populations (200 and 1000) were able to adapt to higher concentrations than before, as expected (Figure S11 A, B). Surprisingly, though, while the 2000 population managed to reach a higher concentration of 18 nM (Figure S11 C), the median is the same (15 nM) as in the original experiment or the smaller population of 1000 individuals (Figure S12). The median value of the 1000-individual population was also 15 nM. However, adaptation was faster at the beginning in populations with 2000 individuals, when compared to the populations with 2000 individuals. This indicates that mainly the initial adaptation is constrained by a small population size when mutation supply is the limiting factor.

(A) Population size of 200 (B) Population size of 1000 (C) Population size of 2000

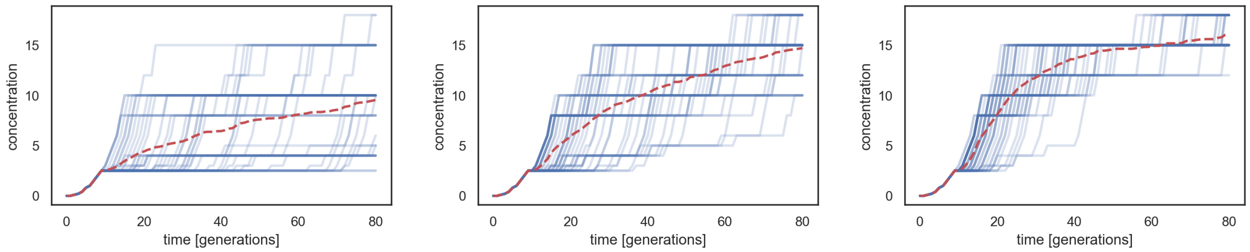

Figure S11: **Concentration at which the worm population grew as a function of time.** Blue solid lines correspond to individual simulations; the red dashed line represents the mean.

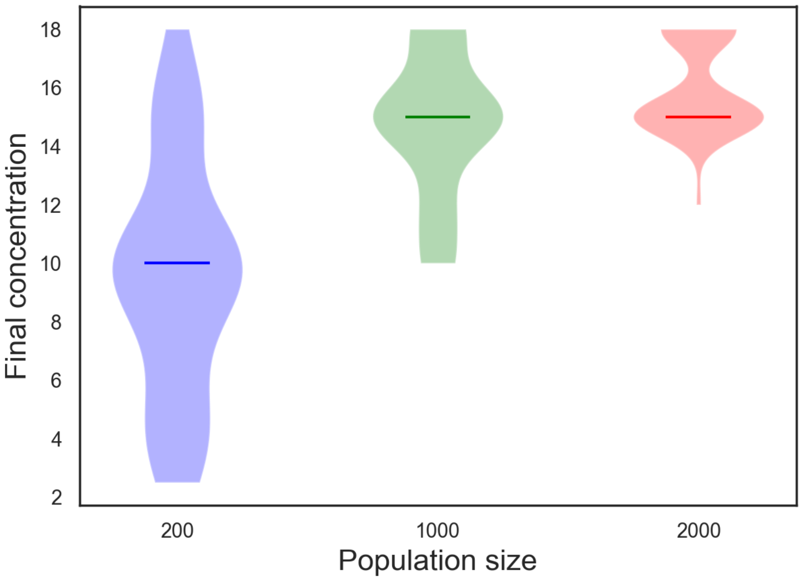

Figure S12: **Distribution of the final concentration reached after 80 generations.** Horizontal lines represent medians.

S2.3.3 Neutral evolution

Figure S13 shows an average number of homozygous mutations per worm present in the population over time in computer simulations modelling neutral evolution in three different populations of worms. The number of homozygous (mutated) loci is very low in all three population sizes. Variance is the highest in the results of simulations of the smallest populations, as expected. This suggests that the number of mutations per worm may not be strongly influenced by the population size under these conditions, and the results observed and described above are caused by the selection pressure created by the drug containing environment.

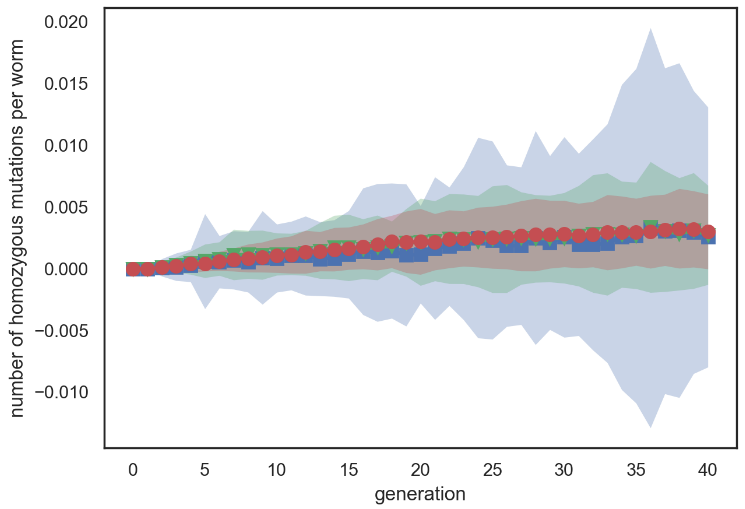

Figure S13: **Simulations of the neutral evolution.**
The average number of homozygous mutations per worm present within the population at a certain time point: 200 (blue), 1000 (green) and 2000 (red).

As the mutations were not beneficial, they reached only a very small frequency, if ever, after 40 generations. Homozygous resistant mutants are almost absent in the final populations of any size S14.

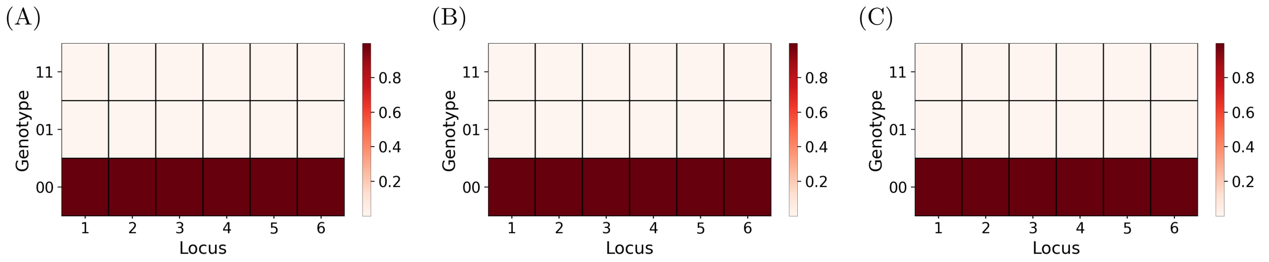

Figure S14: **The average genotype frequencies of 100 final populations.**
Different population sizes: a) 200 b) 1000 c) 2000. On the X axis, different loci are portrayed, and on the Y axis, the genotypes that are possible for each locus. The rectangle's colour corresponds to the fraction of individuals having a particular genotype on that particular locus. Values in each column add to 1.

References

Barbora Trubenová, Jacqueline Hellinga, Jürgen Krücken, Georg Von Samson-Himmelstjerna, Hinrich Schulenburg, and Roland R. Regoes. Investigating the consequences of the mating system for drug resistance evolution in Caenorhabditis elegans. *Proceedings of the Royal Society B: Biological Sciences*, 292(2057), 10 2025. ISSN 14712954. doi: 10.1098/RSPB.2025.1181/234792. URL `https://dx.doi.org/10.1098/rspb.2025.1181`.

Judith Kimble and Samuel Ward. Germ-line Development and Fertilization. *Cold Spring Harbor Monograph Archive*, 17(0):191–213, 1 1988. doi: 10.1101/0.191-213. URL `https://cshmonographs.org/index.php/monographs/article/view/5022`.

Viktoria Wegewitz, Hinrich Schulenburg, and Adrian Streit. Experimental insight into the proximate causes of male persistence variation among two strains of the androdioecious Caenorhabditis elegans (Nematoda). *BMC Ecology*, 8:1–12, 2008. ISSN 14726785. doi: 10.1186/1472-6785-8-12.

Adam K. Bahrami and Yun Zhang. When females produce sperm: Genetics of C. Elegans hermaphrodite reproductive choice. *G3: Genes, Genomes, Genetics*, 3(9):1851–1859, 2013. ISSN 21601836. doi: 10.1534/G3.113.007914/-/DC1/FIGURES4.PDF. URL `https://pmc.ncbi.nlm.nih.gov/articles/PMC3789810/`.

Susan Strome, William G. Kelly, Sevinc Ercan, and Jason D. Lieb. Regulation of the X Chromosomes in Caenorhabditis elegans. *Cold Spring Harbor Perspectives in Biology*, 6(3):a018366, 3 2014. ISSN 19430264. doi: 10.1101/CSHPERSPECT.A018366. URL `https://pmc.ncbi.nlm.nih.gov/articles/PMC3942922/`.

I. Jana I. Janssen, Jürgen Krücken, Janina Demeler, and Georg Von Samson-Himmelstjerna. Caenorhabditis elegans: Modest increase of susceptibility to ivermectin in individual P-glycoprotein loss-of-function strains. *Experimental Parasitology*, 134(2):171–177, 2013. ISSN 00144894. doi: 10.1016/j.exppara.2013.03.005. URL `http://dx.doi.org/10.1016/j.exppara.2013.03.005`.

Antony P. Page. The sensory amphidial structures of Caenorhabditis elegans are involved in macrocyclic lactone uptake and anthelmintic resistance. *International Journal for Parasitology*, 48(13):1035–1042, 2018. ISSN 18790135. doi: 10.1016/j.ijpara.2018.06.003. URL `https://doi.org/10.1016/j.ijpara.2018.06.003`.

Catherine E. James and Mary W. Davey. Increased expression of ABC transport proteins is associated with ivermectin resistance in the model nematode Caenorhabditis elegans. *International Journal for Parasitology*, 39(2):213–220, 2009. ISSN 00207519. doi: 10.1016/j.ijpara.2008.06.009.

Cécile Ménez, Mélanie Alberich, Dalia Kansoh, Alexandra Blanchard, and Anne Lespine. Acquired tolerance to ivermectin and moxidectin after drug selection pressure in the nematode Caenorhabditis elegans. *Antimicrobial Agents and Chemotherapy*, 60(8):4809–4819, 2016. ISSN 10986596. doi: 10.1128/AAC.00713-16.

Mark D. Mathew, Neal D. Mathew, Angela Miller, Mike Simpson, Vinci Au, Stephanie Garland, Marie Gestin, Mark L. Edgley, Stephane Flibotte, Aruna Balgi, Jennifer Chiang, Guri Giaever, Pamela Dean, Audrey Tung, Michel Roberge, Calvin Roskelley, Tom Forge, Corey Nislow, and Donald Moerman. Using C. elegans Forward and Reverse Genetics to Identify New Compounds with Anthelmintic Activity. *PLoS Neglected Tropical Diseases*, 10(10):1–28, 2016. ISSN 19352735. doi: 10.1371/journal.pntd.0005058.

Joseph A. Dent, McHardy M. Smith, Demetrios K. Vassilatis, and Leon Avery. The genetics of ivermectin resistance in Caenorhabditis elegans. *Proceedings of the National Academy of Sciences of the United States of America*, 97(6):2674–2679, 2000. ISSN 00278424. doi: 10.1073/pnas.97.6.2674.

Dee R. Denver, Krystalynne Morris, Michael Lynch, and W. Kelley Thomas. High mutation rate and predominance of insertions in the Caenohabditis elegans nuclear genome. *Nature*, 430(7000):679–682, 8 2004. ISSN 00280836. doi: 10.1038/NATURE02697;KWRD=SCIENCE. URL https://www.nature.com/articles/nature02697.

John Spieth, Daniel Lawson, Paul Davis, Gary Williams, and Kevin Howe. Overview of gene structure in C. elegans. *WormBook : the online review of C. elegans biology*, pages 1–18, 2014. ISSN 15518507. doi: 10.1895/WORMBOOK.1.65.2,. URL https://pubmed.ncbi.nlm.nih.gov/25368915/.
