# Supplementary material for: Evolution of Ivermectin Resistance in the Nematode Model Caenorhabditis elegans : Critical Influence of Population Size and Altered Emodepside Efficacy
Source: Evol Appl. 2026 Apr 24;19(4):e70241. doi: 10.1111/eva.70241 (PMC13108422; doi:10.1111/eva.70241)
Supplement: Supplementary file 2 — Table S1: Genomic mutation rate of evolved populations during the evolution experiment. Table S2:. Increase resistance of C. elegans against ivermectin. Table S3: Mutational effects used in the simulations. Table S4: Sequence of ivermectin concentrations in nM. [file EVA-19-e70241-s001.docx]

**Table S1:Genomic mutation rate of evolved populations during the evolution experiment.**

|  |  |  |
| --- | --- | --- |
| **Population** | **Total number of *de novo* mutations** | **Mutation rate (per site per genome)** |
| 2000R | 4082 | $10.46 x 10^-7^ |
| 2000C | 2860 | $7.33 x 10^-7^ |
| 1000R | 3973 | $10.18 x 10^-7^ |
| 1000C | 2949 | $7.56 x 10^-7^ |
| 200R | 2427 | $6.22 x 10^-7^ |
| 200C | 2447 | $6.27 x 10^-7^ |

**Table S2: Increase resistance of *C. elegans* against ivermectin**

| **gene/strain** | **value** | **fold increase** | **reference** |
| --- | --- | --- | --- |
| N2 | 3.693 nM |  | Janssen et al. [2013] |
| che-1 | lot more than 10 nM |  | Page [2018] |
| che-11 | lot more than 10 nM |  | Page [2018] |
| che-13 | lot more than 10 nM |  | Page [2018] |
| osm-5 | lot more than 10 nM |  | Page [2018] |
| daf-10 | lot more than 10 nM |  | Page [2018] |
| dyf- 3 | lot more than 10 nM |  | Page [2018] |
| dyf-4 | lot more than 10 nM |  | Page [2018] |
| dyf-7 | lot more than 10 nM |  | Page [2018] |
| mec-8 | lot more than 10 nM |  | Page [2018] |
| bbs-1 | lot more than 10 nM |  | Page [2018] |
| N2 parental |  | 1 | James and Davey [2009] |
| IVR6 (multiple mutations) |  | 4.5 | James and Davey [2009] |
| IVR10 (multiple mutations) |  | 19 | James and Davey [2009] |
| wild type (multiple mutations) | EC50 = 1.69 nM |  | Ménez et al. [2016] |
| IVR10 (multiple mutations) | EC50 = 12.43 nM |  | Ménez et al. [2016] |
| normal strain | IC50 = 0.03 $\mu M$ |  | Mathew et al. [2016] |
| DA1316 (several mutations) | IC50 = 7 $\mu M$ |  | Mathew et al. [2016] |
| wt | EC37=1.1 ng/ml |  | Dent et al. [2000] |
| avr-15, UNC-7 | EC37=733 ng/ml |  | Dent et al. [2000] |
| avr-15, avr-14, glc-1 | EC37=4264 ng/ml |  | Dent et al. [2000] |
| avr-15, avr-14 | EC37=13,8 ng/ml |  | Dent et al. [2000] |

**Table S3: Mutational effects used in the simulations**

| **Locus** | **1** | **2** | **3** | **4** | **5** | **6** |
| --- | --- | --- | --- | --- | --- | --- |
| **Benefit** | 0.5 | 0.5 | 0.5 | 3 | 3 | 3 |
| **Cost** | 0.05 | 0.05 | 0.05 | 0.1 | 0.2 | 0.3 |

**Table S4: Sequence of ivermectin concentrations in nM.**

| 0 | 0.1 | 0.2 | 0.4 | 0.8 | 1 | 1.5 | 2 | 2.5 | 3 | 4 | 5 | 6 | 8 | 10 | 12 | 15 | 18 | 21 | 24 | 28 | 32 | 36 | 40 | 45 |  |
| --- | --- | --- | --- | --- | --- | --- | --- | --- | --- | --- | --- | --- | --- | --- | --- | --- | --- | --- | --- | --- | --- | --- | --- | --- | --- |
